# The transcriptional repressor BLIMP1 enforces TCF-1-dependent and -independent restriction of the memory fate of CD8^+^ T cells

**DOI:** 10.1016/j.immuni.2025.09.008

**Published:** 2025-10-02

**Authors:** Maegan K. Murphy, Matthew McCullen, Joshua L. Deffenbaugh, Andy Y. Chen, Joy Pai, Bence Daniel, Amir Yousif, Saravanan Raju, Sunnie Hsiung, Zhenxiao Wang, Hazem E. Ghoneim, Ansuman T. Satpathy, Marco Colonna, Eugene M. Oltz, Takeshi Egawa

**Affiliations:** 1 Department of Pathology and Immunology, Washington University School of Medicine, St. Louis, MO 63110, USA; 2 Department of Microbial Infection and Immunity, The Ohio State University College of Medicine, Columbus, OH, USA; 3 Department of Pathology, Stanford University, Stanford, CA, USA; 4 Department of Bioengineering, Stanford University, Stanford, CA, USA; 5 Department of Immunology, Stanford University, Stanford, CA, USA; 6 Parker Institute for Cancer Immunotherapy, San Francisco, CA, USA; 7 Stanford Cancer Institute, Stanford University, Stanford, CA, USA; 8 Gladstone-UCSF Institute of Genomic Immunology, San Francisco, CA, USA; 9 Pelotonia Institute for Immuno-Oncology, The Ohio State University Comprehensive Cancer Center, Columbus, OH, USA; 10 Present address: Calico Life Sciences, South San Francisco, CA, USA; 11 Present address: Genentech Inc., South San Francisco, CA, USA; 12 Present address: Department of Pathology, University of California, San Diego, La Jolla, CA, USA; 13 Lead contact

## Abstract

During differentiation of CD8^+^ T cells, the transcription factors TCF-1 and Blimp1 control progenitor and terminally differentiated states, respectively. Here, we examined the hierarchy and functional consequences of cross-regulation between these factors. We identified two Blimp1-bound *cis*-regulatory elements, *Tcf7*^+22kb^ and *Tcf7*^+17kb^, that enforced *Tcf7* silencing in a context-specific manner during both acute and chronic responses. Deletion of these elements decoupled *Tcf7* repression from effector differentiation but did not rewire effector T cells to a memory state or prevent the acquisition of phenotypic hallmarks of exhaustion. However, combined ablation of *Prdm1* and *Tcf7* preserved a memory surface phenotype despite defects in secondary expansion. Thus, the anti-proliferative and pro-differentiative effects of Blimp1 in effector or exhausted CD8^+^ T cells represent mechanistically distinct modules, wherein repression of *Tcf7* limits proliferative capacity but not memory or progenitor specification.

## In brief

TCF-1, encoded by *Tcf7*, supports CD8^+^ T cell memory and is silenced in effector T cells. Murphy et al. identify two *cis*-elements required for Blimp1-mediated *Tcf7* repression and find that decoupling *Tcf7* silencing from effector differentiation is permissive for effector gene expression. Thus, Blimp1 restricts memory differentiation through both *Tcf7*-dependent and -independent pathways.

## Graphical Abstract

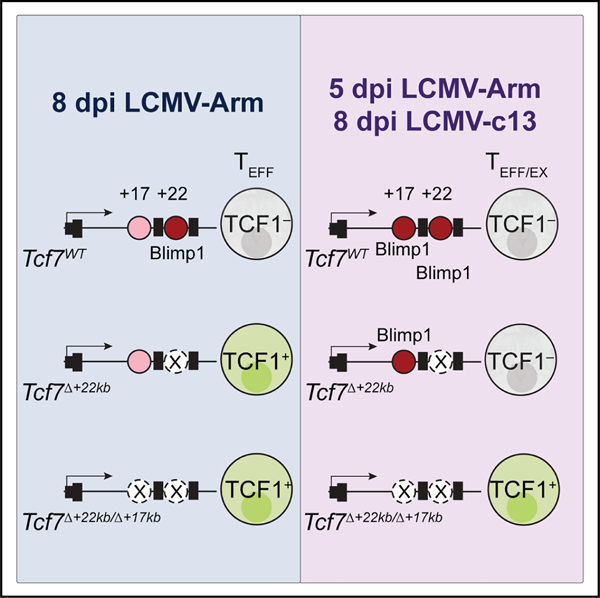

## INTRODUCTION

CD8^+^ T cells eradicate intracellular pathogens and tumors through cytotoxic functions. Upon antigen recognition, naive CD8^+^ T cells clonally expand and differentiate to a cytotoxic phenotype. Some persist as memory cells in a quiescent state, lack cytotoxicity, and express naive cell markers such as CD62L and CD127, while most effector cells die.^1–4^ In contrast to transient antigen exposure, antigen persistence elicits altered responses by CD8^+^ T cells, which acquire an “exhaustion” phenotype. Following initial expansion in chronic infection, CD8^+^ T cells are maintained for weeks or months at attenuated levels.^5–10^ Chronic effector responses are maintained by progenitor exhausted T (Tpex) cells, which remain undifferentiated and retain proliferative potential.^11,12^ Tpex cells respond to PD-1 immune checkpoint blockade^11,12^ and are of interest in vaccine development and anti-tumor therapies.

Both memory CD8^+^ T cells and Tpex cells express the transcription factor (TF) TCF-1, encoded by *Tcf7*. *Tcf7* is expressed throughout T cell development and is downregulated in terminally differentiated CD8^+^ T cells that upregulate the transcriptional repressor Blimp1.^13^ *Tcf7* is required for both Tpex differentiation and memory T cell recall capacity.^11,12,14^ Conversely, overexpression of TCF-1 reinforces the maintenance of progenitor-like CD8^+^ T cell states.^15^ Antigen-specific CD8^+^ T cells that lose *Tcf7* expression at the peak of an acute response fail to contribute to memory CD8^+^ T cells.^13^ Although the loss of *Tcf7* expression in effector CD8^+^ T cells may limit longevity, the mechanisms by which *Tcf7* is silenced in response to antigen or cytokine signaling remain undefined.

Blimp1 (encoded by *Prdm1*) has been implicated in antagonizing memory cell differentiation of both CD4^+^ and CD8^+^ T cells, potentially through direct repression of *Tcf7*.^16–20^ However, de-repression of *Tcf7* in Blimp1-deficient cells may be secondary to increases in expression of *Bcl6*.^19^ Moreover, TCF-1 represses *Prdm1* in several cellular contexts.^16,18,21^ These results collectively indicate that mutual antagonism of TCF-1 and Blimp1 sets a threshold for effector differentiation. However, both how the balance of TCF-1 and Blimp1 regulates T cell fate and the hierarchical interactions of these factors remain important questions.

Here, we identified an intronic silencer in the *Tcf7* locus that becomes accessible in differentiated CD8^+^ T cells. Deletion of this element or mutagenesis of its Blimp1 binding site resulted in retention of TCF-1 in effector T cells. Additional mutagenesis of a neighboring Blimp1-bound region further enhanced TCF-1 de-repression in the presence of antigen. Decoupling downregulation of TCF-1 from effector differentiation exerted subtle reductions in effector gene expression but failed to redirect effector cells to a memory fate. Importantly, Blimp1 deficiency bypassed the requirement for TCF-1 in the specification of Tpex, while CD8^+^ T cells lacking both TFs were defective in secondary expansion. We conclude that Blimp1 restricts the memory fate through targeting both TCF-1-dependent and -independent programs.

## RESULTS

### *Tcf7*^+22kb^ mediates *Tcf7* downregulation by IL-2 and IL-12 receptor signaling

**Figure 1.**
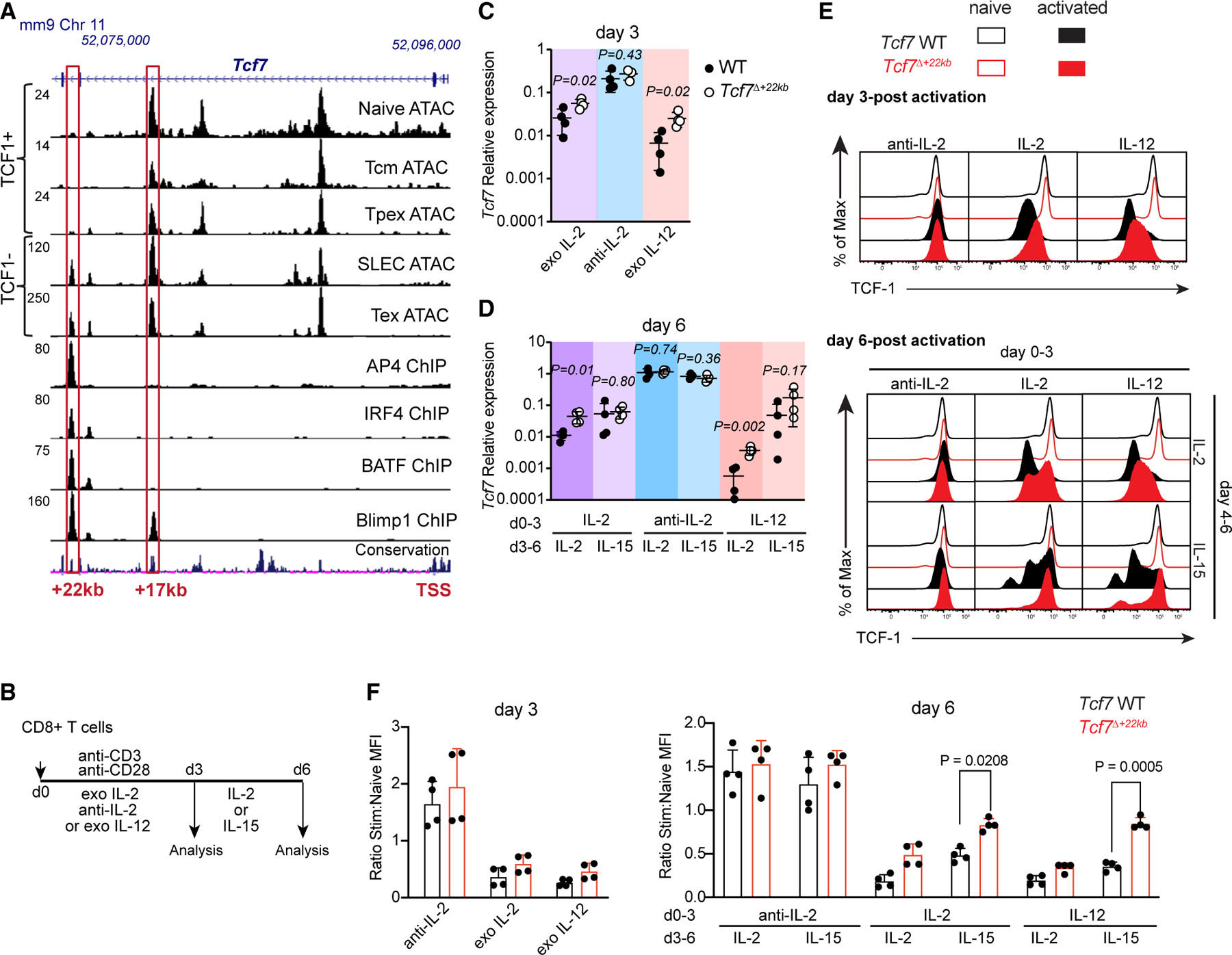
Ablation of *Tcf7*^+22kb^ limits IL-2 and IL-12-dependent downregulation of *Tcf7 in vitro* (A) Analysis of ChIP (GEO: GSE58075, GSE85172, GSE79339) and ATAC (GEO: GSE88987) data of the *Tcf7* locus in CD8^+^ T cells. (B) Experimental approach corresponding to (C)–(E). (C and D) Relative *Tcf7* transcript levels normalized to *Hprt1* in WT (closed circles) or *Tcf7*^Δ+22kb^ (open circles) CD8^+^ T cells stimulated for 3 days (C) and subsequently rested for 3 days in 100 U/mL IL-2 or 10 ng/mL IL-15 (D). *n* = 2 in 2 independent experiments. Data are shown as mean ± SD. Unpaired *t* tests. (E) Representative TCF-1 protein staining of WT (black) and *Tcf7*^Δ+22kb^ (red) CD8^+^ T cells at indicated time points, representative of two experiments conducted in duplicate. Solid histograms represent post-stimulation conditions, and open histograms represent naive CD8^+^ T cells. (F) Quantitation of the ratio of stimulated to naive TCF-1 MFI. Data are pooled from two experiments conducted in duplicate, represented as mean ± SD. Two-way ANOVA and Sidak’s multiple comparison test. See also Figure S1.

To identify regulatory elements involved in *Tcf7* silencing, we analyzed chromatin accessibility surrounding the *Tcf7* locus using published assay for transposase-accessible chromatin using sequencing (ATAC-seq) and chromatin immunoprecipitation sequencing (ChIP-seq) datasets of naive, memory, and terminally differentiated effector (SLEC) CD8^+^ T cells (GEO: GSE58075, GSE85172, GSE7933, GSE88987). We additionally performed ATAC-seq on Tpex and TIM3^+^ exhausted CD8^+^ T cells (Tex) from *Prdm1*-YFP reporter mice, defining Tpex as TIM3^−^ YFP^−^ and Tex as TIM3^+^ YFP^+^ (Figure 1A).^18,22^ Among several evolutionarily conserved accessible chromatin regions (DARs), we identified an intronic region approximately +22 kb from the transcription start site (TSS), termed *Tcf7*^+22kb^. Accessibility of this region correlated inversely with *Tcf7* expression. Additionally, *Tcf7*^+22kb^ is bound by effector-related TFs, including IRF4 and BATF complexes that bind to AP-1/IRF composite binding elements (AICEs)^23^; TFAP4, which binds to E-box sequences; and Blimp1.^24,25^

We interrogated the role of *Tcf7*^+22kb^ in transcriptional activity of the locus by generating mice lacking a ∼250 bp sequence in *Tcf7*^+22kb^ (Figure S1A). To determine whether *Tcf7*^+22kb^ was required for *Tcf7* downregulation, CD8^+^ T cells from wild-type (WT) and *Tcf7*^Δ+22kb^ mice were activated *in vitro* to model effector or memory differentiation.^26^ Purified splenic CD8^+^ T cells were stimulated with anti-CD3/CD28 in the presence of either interleukin-2 (IL-2), anti-IL-2, or IL-12, followed by 3 days of resting in IL-2 or IL-15 to determine the kinetics of *Tcf7* downregulation (Figure 1B). In the initial 3-day culture, *Tcf7* mRNA was highest in anti-IL-2 culture conditions (Figure 1C). Exogenously added IL-2 and IL-12 downregulated *Tcf7* mRNA in WT cells relative to anti-IL-2 conditions within 3 days of activation. *Tcf7*^Δ+22kb^ CD8^+^ T cells retained 2- or 3-fold higher levels of *Tcf7* mRNA than WT cells in the presence of IL-2 or IL-12. These differences became more pronounced following resting in IL-2 (Figure 1D). Resting cultures with IL-15 maintained higher levels of *Tcf7* compared with IL-2 rested cells, consistent with the roles of these cytokines in effector vs. memory differentiation *in vitro*.^26^ Consistently, CD8^+^ T cells primed in the absence of IL-2R signaling retained TCF-1 protein independently of the *Tcf7*^+22kb^ element (Figure 1E). However, larger proportions of *Tcf7*^Δ+22kb^ CD8^+^ T cells retained TCF-1 after priming in the presence of IL-2 or IL-12, particularly following 3 days of resting. Normalization of mean fluorescence intensity (MFI) of TCF-1 in activated T cells to the MFI of naive cells revealed that TCF-1 expression was downregulated on day 3 of both IL-2 and IL-12 culture irrespective of *Tcf7*^+22kb^ genotype, but *Tcf7*^Δ+22kb^ CD8^+^ T cells rested in IL-15 substantially restored TCF-1 expression relative to WT controls (Figure 1F). Consequently, *Tcf7*^+22kb^ is a silencer of *Tcf7* expression in CD8^+^ T cells after co-activation of the T cell receptor (TCR) and IL-2R or IL-12R and is particularly important for maintaining TCF-1 expression after withdrawal of TCR stimulation.

### Blimp1 directly represses *Tcf7* in CD8^+^ T cells *in vitro*

**Figure 2.**
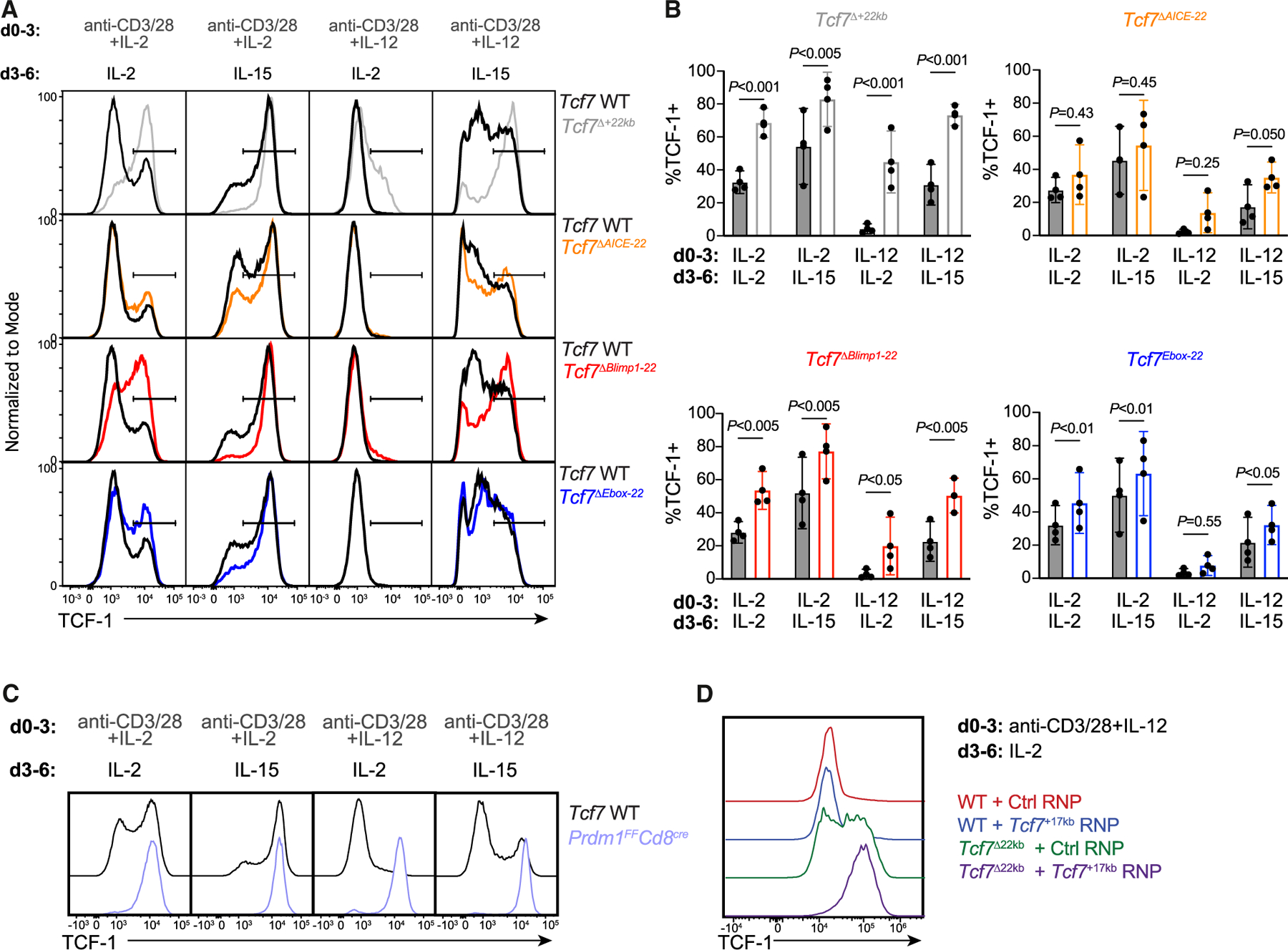
Blimp1 directly antagonizes *Tcf7* expression in CD8^+^ T cells *in vitro* (A and B) (A) TCF-1 protein levels and (B) TCF-1^+^ cell frequencies in co-cultured WT (filled columns) and *Tcf7*^Δ+22kb^ mutant (open columns) CD8^+^ T cells were stimulated for 3 days with anti-CD3/28 with either 100 U/mL IL-2 or 10 ng/mL IL-12 and subsequently rested for 3 days in 100 U/mL IL-2 or 10 ng/mL IL-15. Data from three experiments are shown with mean ± SD. Two-way ANOVA and Sidak’s multiple comparisons test. (C) TCF-1 levels in co-cultured WT and *Prdm1^F/F^ Cd8^cre^* CD8^+^ T cells on day 6 of culture in the indicated conditions. Representative of a technical duplicate from one experiment. (D) TCF-1 expression in WT and *Tcf7*^Δ+22kb^ T cells electroporated with *Cd19* or *Tcf7*^+17kb^ targeting Cas9-sgRNA complexes and cultured 3 days with anti-CD3/28 with 10 ng/mL IL-12 followed by resting 3 days in 100 U/mL IL-2. Data are representative of two experiments. See also Figure S1.

Blimp1, BATF, IRF4, and TFAP4 contribute to effector differentiation in activated CD8^+^ T cells.^25,27–30^ To define the requirement for direct binding of these factors to the *Tcf7*^+22kb^ silencer (Figure S1B), we specifically mutated each of the binding sites in the mouse germline. These alleles were respectively referred to as *Tcf7*^*ΔBlimp1−*22^, *Tcf7*^*ΔAICE−22*^, and *Tcf7*^*ΔEbox-22*^. ChIP-qPCR validated the lack of IRF4 binding in *Tcf7*^*ΔAICE−22*^ cells (Figure S1C). *Tcf7*^*ΔBlimp1−22*^ cells recapitulated the effect of *Tcf7*^+22kb^ deletion on TCF-1 expression in most culture conditions (Figures 2A and 2B). However, activation in IL-12 followed by resting in IL-2 resulted in higher retention of TCF-1 in *Tcf7*^Δ+22kb^ CD8^+^ T cells but modest TCF-1 retention in *Tcf7*^*ΔBlimp1−22*^ cells. Despite more moderate effects, *Tcf7*^*ΔAICE−22*^ and *Tcf7*^*ΔEbox−22*^ CD8^+^ T cells tended to retain higher TCF-1 expression. These results suggest that Blimp1, which is induced by IL-2R and IL-12R signaling,^31,32^ predominantly enforces *Tcf7*^+22kb^ silencer-dependent repression of *Tcf7*.

Since we observed only a partial rescue of TCF-1 expression in *Tcf7*^Δ+22kb^ CD8^+^ T cells and an attenuated effect in the *Tcf7*^*ΔBlimp1−22*^ mutant, we wondered if IL-12 mediated Blimp1-independent downregulation. However, *Prdm1*-deficient CD8^+^ T cells failed to downregulate TCF-1 expression in all *in vitro* conditions (Figure 2C). An additional region was bound by Blimp1 +17 kb of the TSS (Figure 1A; *Tcf7*^+17kb^). We therefore tested individual and combined requirements of *Tcf7*^+22kb^ and *Tcf7*^+17kb^ by targeting *Tcf7*^+17kb^ with Cas9-single-guide RNA (sgRNA) complexes in WT or *Tcf7*^Δ+22kb^ CD8^+^ T cells. While the single ablation of *Tcf7*^+17kb^ had no impact on TCF-1 expression, deletion of *Tcf7*^+17kb^ in *Tcf7*^Δ+22kb^ CD8^+^ T cells (referred to as *Tcf7*^Δ+17kb/+22kb^) prevented TCF-1 loss following IL-12 culture (Figure 2D). Thus, the *Tcf7*^+17kb^ silencer can compensate for *Tcf7*^+22kb^ deficiency in IL-12- and Blimp1-dependent repression of *Tcf7 in vitro*.

### The *Tcf7*^+22kb^ silencer enforces downregulation of *Tcf7* in CD8^+^ T cells during acute infection

**Figure 3.**
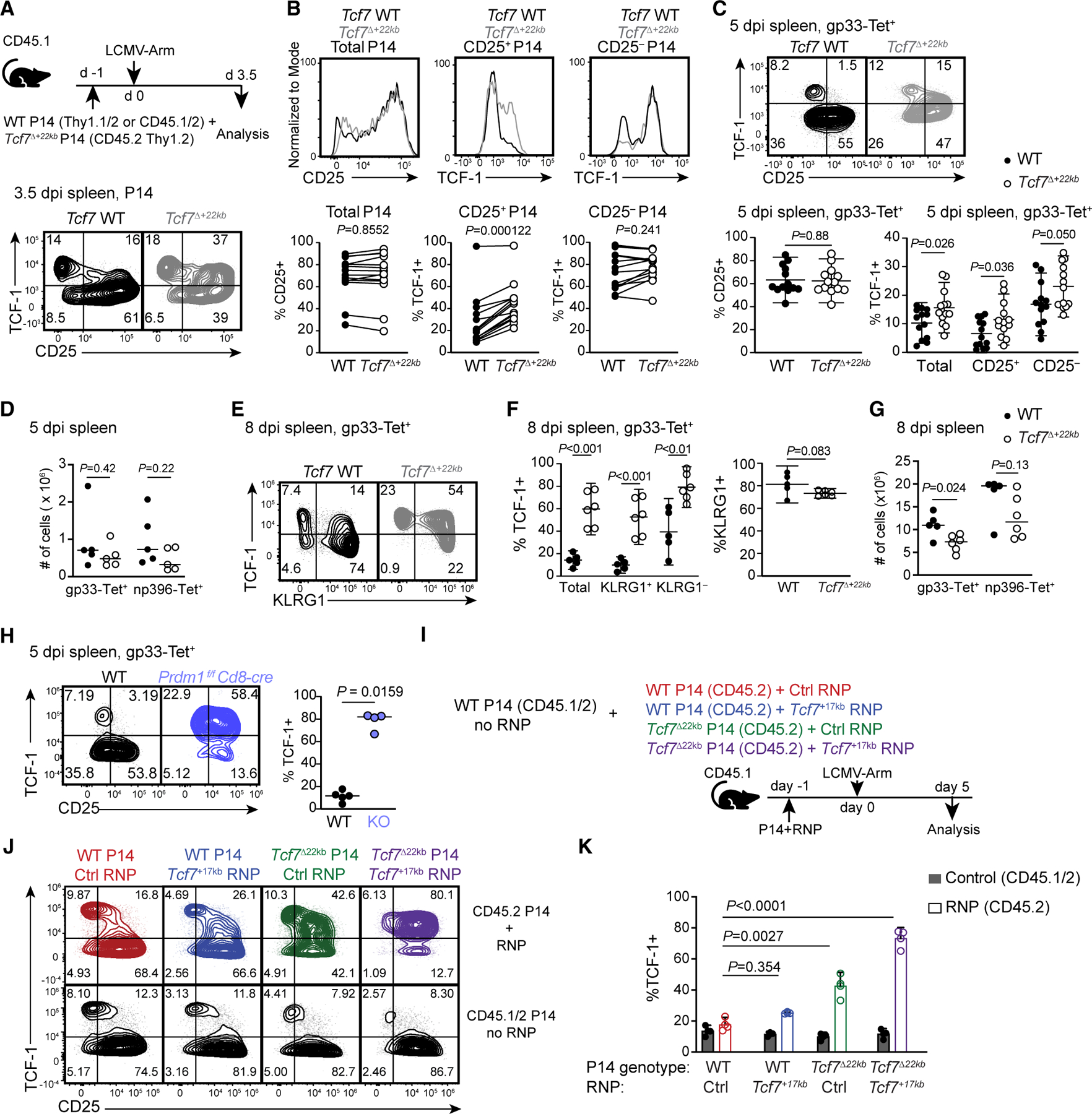
*Tcf7*^+22kb^ enforces the continuous downregulation of *Tcf7* in acute LCMV infection (A) Experimental setup (top) and expression of TCF-1 and CD25 in adoptively transferred WT and *Tcf7*^Δ+22kb^ P14 cells on 3.5 dpi (bottom). (B) Histograms (top) and quantification (bottom) of CD25^+^ cells in P14 cells and TCF-1^+^ cells in CD25^+^ and CD25^−^ cells in donor WT (closed circles) and *Tcf7*^Δ+22kb^ (open circles) P14 cells on 3.5 dpi. Data were pooled from three experiments (*n* = 4–5 per experiment), and statistical differences were assessed by paired Wilcoxon tests. (C) Representative flow plots showing expression of TCF-1 and CD25 by endogenous gp33-specific CD8^+^ T cells in spleens of WT and *Tcf7*^Δ+22kb^ mice on 5 dpi. Data from five experiments are shown as mean ± SD (*n* = 13 per genotype). Unpaired *t* tests with Welch correction. (D) Total numbers of gp33- and np396-specific CD8^+^ T cells in spleens of LCMV-Arm infected mice on 5 dpi, pooled from two experiments (*n* = 5 per genotype), shown with median, and analyzed by the Mann-Whitney test. (E) Representative flow plots showing expression of TCF-1 and KLRG1 by endogenous gp33-specific CD8^+^ T cells in spleens of WT and *Tcf7*^Δ+22kb^ mice on 8 dpi. (F) Frequencies of TCF-1^+^ cells in total gp33-specific, KLRG1^+^, and KLRG1^−^ cells (left) and KLRG1^+^ cells of gp33-specific CD8^+^ T cells with indicated genotypes, pooled from two experiments (*n* = 5 WT and 6 *Tcf7*^Δ+22kb^), and shown as mean ± SD with *p* values by unpaired *t* tests. (G) Numbers of gp33- and np396-specific CD8^+^ T cells in WT and *Tcf7*^Δ+22kb^ mice on 8 dpi with LCMV-Arm, shown as medians and analyzed by the Mann- Whitney test (*n* = 5 WT and 6 *Tcf7*^Δ+22kb^). (H) Representative flow plots showing expression of TCF-1 and CD25 by endogenous gp33-specific CD8^+^ T cells in spleens of WT and *Prdm1*^−/−^ mice on 5 dpi with LCMV-Arm. Data represented as the median of data from two experiments (*n* = 5 WT and 4 *Prdm1*^−/−^ mice). Mann-Whitney test. (I) Schematic of experiment for inhibition of *Tcf7*^+22kb^ and *Tcf7*^+17kb^ elements by electroporation of Cas9/sgRNA RNPs into WT and *Tcf7*^Δ+22kb^ P14 cells. (J) Representative flow plots of adjacent internal control (WT unelectroporated) and corresponding combinations of P14 genotype and RNP in P14 cells isolated from the spleen on 5 dpi with LCMV-Arm. (K) Percentage of P14 cells of each genotype and RNP combination retaining TCF-1 expression on 5 dpi with LCMV-Arm (*n* = 3, representative of three experiments). Data shown as mean ± SD and analyzed by one-way ANOVA and Dunnett’s multiple comparisons test. See also Figure S2.

After validating *Tcf7*^+22kb^ *in vitro*, we interrogated the kinetics of TCF-1 downregulation effector T cells following Lymphocytic Choriomeningitis Virus-Armstrong (LCMV-Arm) infection. Due to the paucity of antigen-specific cells, we transferred naive P14 TCR transgenic CD8^+^ T cells to congenic mice prior to LCMV-Arm challenge to assess TCF-1 expression on 3.5 days post-infection (dpi). WT P14 cells downregulated TCF-1 in CD25^+^ early effector cells (Figures 3A and 3B).^33^ This correlation was clearer in endogenous LCMV-specific CD8^+^ T cells on 5 dpi, in which a vast majority of CD25^+^ cells became TCF-1^−^ (Figure 3C). Down-regulation of TCF-1 was attenuated in *Tcf7*^Δ+22kb^ P14 cells on 3.5 dpi and in endogenous CD8^+^ T cells on 5 dpi (Figures 3B and 3C). However, even when *Tcf7*^+22kb^ was ablated, most CD25^+^ *Tcf7*^Δ+22kb^ CD8^+^ T cells downregulated TCF-1 on 5 dpi.

In contrast to earlier time points, a majority of antigen-specific *Tcf7*^Δ+22kb^ CD8^+^ T cells expressed substantially higher levels of TCF-1 than WT cells on 8 dpi in the spleen, blood, and liver (Figures 3E, 3F, S2A, and S2B). Approximately 20% of splenic gp33-specific CD8^+^ T cells in WT mice expressed TCF-1. However, higher proportions (∼70%) of gp33-specific CD8^+^ T cells in *Tcf7*^Δ+22kb^ mice were TCF-1^+^, including KLRG1^+^ effectors that normally lack TCF-1. Absolute numbers of gp33-specific CD8^+^ T cells were reduced by 35% in *Tcf7*^Δ+22kb^ mice on 8 dpi, although not on 5 dpi or for the np396 response at either time point (Figures 3D and 3G). We additionally confirmed that TCF-1 was retained in *Tcf7*^Δ+22kb^ effector cells in the spleen following infection with *Listeria monocytogenes* expressing an LCMV-gp33 epitope (LM-GP) (Figure S2C).

The incomplete retention of TCF-1 in *Tcf7*^Δ+22kb^ mice on 5 dpi prompted us to evaluate whether the *Tcf7*^+17kb^ element contributed to *Tcf7* silencing. Since most *Prdm1-*deficient CD8^+^ T cells retained TCF-1 on 5 dpi (Figure 3H), we hypothesized that Blimp1 acted on the *Tcf7*^+17kb^ region at this time point. We disrupted *Tcf7*^+17kb^, *Tcf7*^+22kb^, or both by electroporating Cas9-sgRNA complexes targeting *Tcf7*^+17kb^ into naive WT or *Tcf7*^Δ+22kb^ P14 cells and transferred them into congenic mice, which were subsequently infected with LCMV-Arm (Figure 3I). TCF-1 remained highly expressed in a majority of *Tcf7*^Δ+17kb/+22kb^ P14 cells on 5 dpi, which de-repressed *Tcf7* to a greater extent than cells lacking either element alone (Figures 3J and 3K).

Given our *in vitro* findings that IL-12R signaling supplements *Tcf7*^+22kb^-dependent *Tcf7* silencing through *Tcf7*^+17kb^, we sought to determine whether *Tcf7*^+22kb^ and IL-12 redundantly repress *Tcf7* following LM-GP infection, which elicits IL-12 production.^34^ Indeed, combined deficiency of *Tcf7*^+22kb^ and *Il12rb2* resulted in improved TCF-1 retention relative to either single deficiency (Figures S2D and S2E). These results collectively show that the Blimp1-bound *Tcf7*^+22kb^ and *Tcf7*^+17kb^ redundantly facilitate TCF-1 downregulation in CD8^+^ T cells during the early phase of effector differentiation.

### *Tcf7* de-repression is permissive for effector differentiation

**Figure 4.**
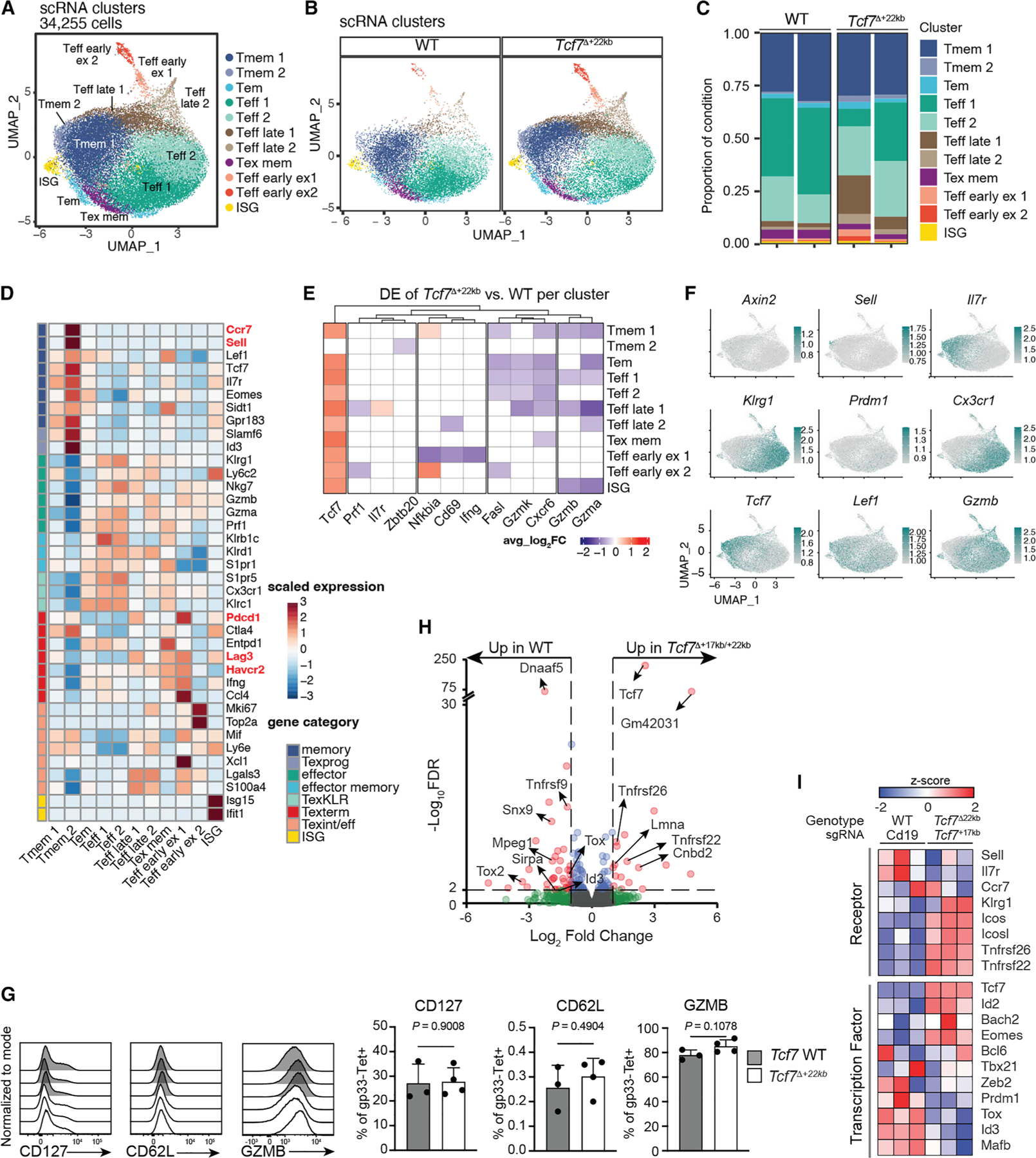
scRNA-seq of WT and *Tcf7*^Δ+22kb^ CD8^+^ T cells in acute infection (A) UMAP plot of gp33-apecific CD8^+^ T cells sorted from two WT and two *Tcf7*^Δ+22kb^ mice 8 dpi with LCMV-Arm. Colored by cluster identity. (B) WT and *Tcf7*^Δ+22kb^ cells displayed separately on UMAP projection. (C) Stacked bar plot of frequency of each cluster in each replicate of gp33-specific CD8^+^ T cells. (D) Heatmap of genes differentially expressed between clusters. (E) Heatmap of genes differentially expressed in WT and *Tcf7*^Δ+22kb^ cells within each cluster. (F) Feature plots of selected genes. (G) Expression of GZMB, CD127, and CD62L analyzed by flow cytometry in gp33-specific CD8^+^ T cells from spleens of WT and *Tcf7*^Δ+22kb^ mice 8 dpi with LCMV-Arm. Data represent two experiments shown as mean ± SD and analyzed by *t* test. (*n* = 3 WT and 4 *Tcf7*^Δ+22kb^ mice). (H) Volcano plot of bulk RNA-seq results from WT/*Cd19* RNP and *Tcf7*^Δ+22kb^/*Tcf7*^+17kb^ RNP P14 cells sorted as KLRG1^+^ on 8 dpi with LCMV-Arm WT/*Cd19* RNP and *Tcf7*^*+*17kb^ RNP (*n* = 3). Cutoffs by adjusted false discovery rate (FDR) < 0.01 and fold-change > 2. (I) Heatmap of selected receptors and TFs in RNA-seq of KLRG1^+^ WT/*Cd19* RNP and *Tcf7*^Δ+22kb^/*Tcf7*^+17kb^ RNP P14 cells of 8 dpi LCMV-Arm. See also Figure S3.

To address the impact of decoupling TCF-1 loss from effector differentiation on gene regulation, we conducted single-cell (sc)RNA- and scATAC-seq on splenic gp33-specific CD8^+^ T cells from WT and *Tcf7*^*Δ*+22kb^ mice on 8 dpi with LCMV-Arm, since *Tcf7*^*Δ*+22kb^ effector cells de-repressed *Tcf7* at this time. From scRNA-seq, we identified eleven clusters associated with memory, effector memory, effector (Teff), early exhausted, and interferon (IFN)-stimulated gene (ISG)-enriched signatures (Figure 4A). WT and *Tcf7*^Δ+22kb^ CD8^+^ T cells were similarly represented in most clusters, although deletion of the *Tcf7*^+22kb^ silencer was associated with enrichment of the Tmem2 cluster, marked by expression of *Sell* and *Ccr7* (Figures 4B–4F). *Tcf7*^Δ+22kb^ CD8^+^ T cells exhibited enrichment of the Teff late 1 cluster, which expressed the inhibitory receptors *Lag3*, *Havcr2*/TIM3, and *Pdcd1*/PD-1 (Figure 4D). Analysis of pseudo-bulk gene expression data in each cluster revealed increased *Tcf7* expression in *Tcf7*^Δ+22kb^ cells in all clusters except for Tmem2 (Figure 4E). Conversely, expression of effector-related genes, including *Gzmb*, *Gzma*, *Fasl*, and *Ifng*, was reduced in *Tcf7*^Δ+22kb^ cells in several effector clusters, although expression of GZMB protein was comparable between WT and *Tcf7*^Δ+22kb^ cells (Figures 4E, 4G, and S3A). We additionally profiled *Tcf7*^Δ+22kb^ cells for expression of CD62L and CD127 but did not observe differences from WT cells (Figure 4G). Therefore, retention of TCF-1 attenuates expression of several genes implicated in cytotoxicity but is conducive to many features of an effector phenotype.

The incomplete *Tcf7* de-repression in *Tcf7*^Δ+22kb^ CD8^+^ T cells and transcript dropout in the scRNA-seq dataset may have limited detection of de-repressed genes in TCF-1^+^ effector cells. We therefore conducted bulk RNA-seq on WT and *Tcf7*^Δ+17kb/+22kb^ P14 cells on 8 dpi with LCMV-Arm. Analysis revealed limited differences in gene expression, with 23 genes upregulated and 52 genes downregulated in *Tcf7*^Δ+17kb/+22kb^ P14 cells (Figure 4H). Several TFs were downregulated, including *Tox*, *Tox2*, and *Id3* (Figures 4H and 4I). Excepting *Tcf7*, memory-associated genes were not upregulated in *Tcf7*^Δ+17kb/+22kb^ cells (Figure 4I). These data, consistent with the scRNA-seq results, affirm that *Tcf7* retention modestly impairs effector differentiation and is insufficient to divert effectors to a memory precursor (MPEC) state.

The scATAC-seq analysis highlighted unique clustering of WT and *Tcf7*^Δ+22kb^ cells (Figures S3B and S3C). To infer cluster identities, gene activity scores were calculated as weighted averages of the accessibility signal around the TSS of each gene, serving as a surrogate for gene expression.^35^ *Tcf7* and *Id3* gene activity scores were enriched in clusters in which *Tcf7*^Δ+22kb^ cells were more prevalent (Figures S3C and S3D). Gene activity scores for *Il7r* and *Klrg1*, which delineate MPEC and SLEC populations, respectively, were similarly enriched in WT- and *Tcf7*^Δ+22kb^-dominated clusters. We asked whether *Tcf7*^+17kb^ accessibility differed between clusters, which could account for its context-specific redundancy with *Tcf7*^+22kb^. Indeed, *Tcf7*^+17kb^ accessibility was most abundant in *Tcf7*^Δ+22kb^-enriched regions of the uniform manifold approximation and projection (UMAP) in clusters 7, 8, and 11, in which *Tcf7*^+22kb^ was predicted to be inaccessible based on population-based analysis (Figure S3E). These results suggest that *Tcf7*^+17kb^ is constitutively accessible in *Tcf7*^hi^ cells and acts compensatorily in *Tcf7*^Δ+22kb^ cells.

We next conducted motif enrichment analysis to infer whether TCF-1 retention impacted accessibility of chromatin to TF binding (Figure S3F). Increased accessibility of BATF, TCF-1, and TBX1 binding motifs and decreased accessibility of FLI1 motifs were observed in clusters that were dominantly composed of *Tcf7*^Δ+22kb^ cells. Despite the de-repression of *Tcf7* in *Tcf7*^Δ+22kb^ effectors, TCF-1 motifs remained under-represented in effector clusters even where *Tcf7*^Δ+22kb^ cells predominated. These data suggest that epigenetic states associated with MPECs are prerequisites for TCF-1 activity on transcriptional targets that promote CD8^+^ T cell memory. Consistently, *Tcf7* de-repression is permissive for the generation of an effector response, perhaps because pro-effector factors reduce the chromatin accessibility of TCF-1 binding sites even when TCF-1 remains expressed.

### Retention of *Tcf7* by deletion of *Tcf7*^+22kb^ is insufficient to promote memory differentiation

**Figure 5.**
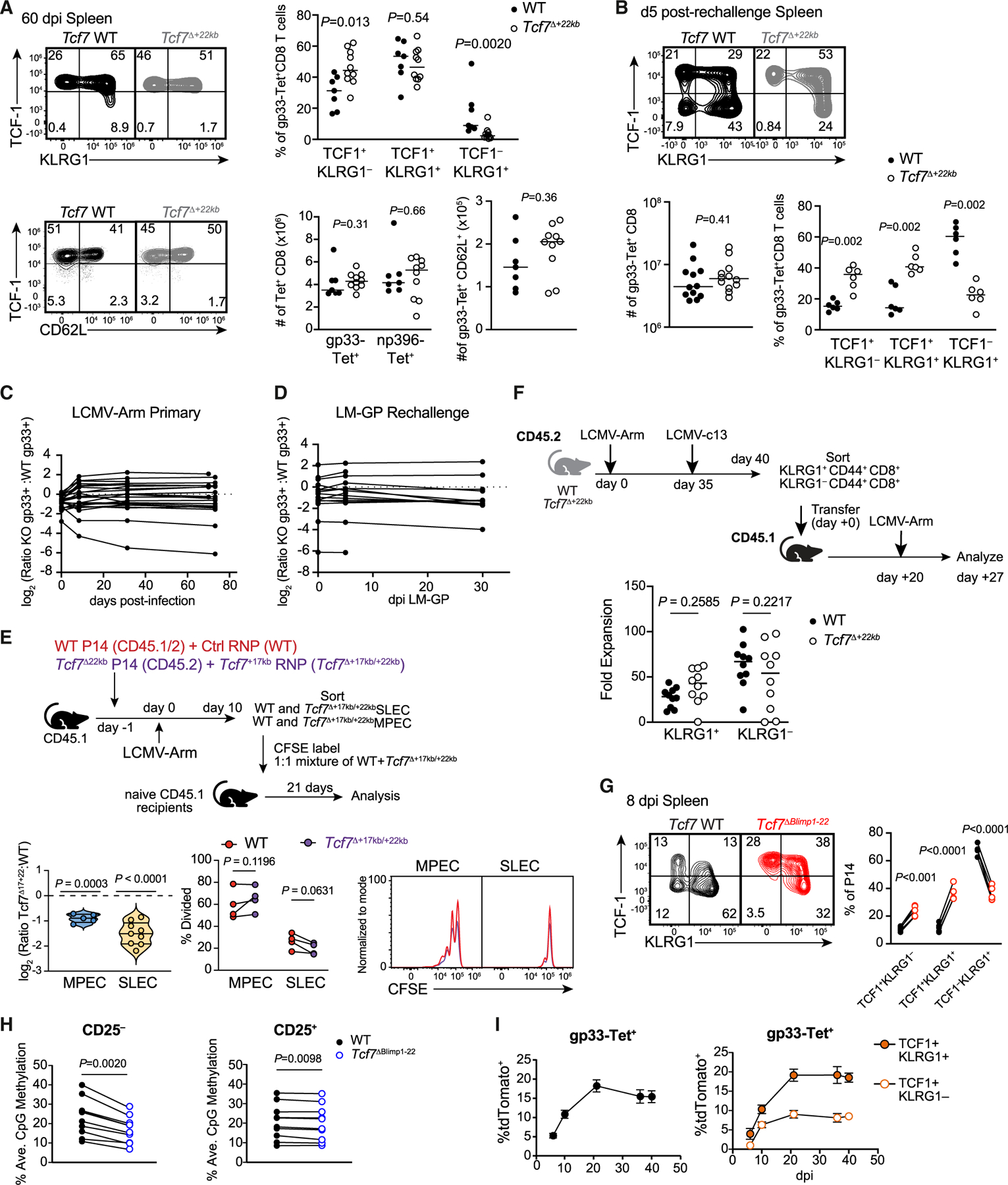
Direct binding of Blimp1 to *Tcf7*^+22kb^ restricts *Tcf7* in CD8^+^ T cells in acute infection (A) Representative flow plots and statistical analysis of total numbers and phenotypic frequencies of gp33-specific specific WT and *Tcf7*^Δ+22kb^ CD8^+^ T cells on 60 dpi with LCMV-Arm infection. Data from two experiments (*n* = 7 WT and 10 *Tcf7*^Δ+22kb^) are shown with medians and analyzed by Mann-Whitney tests. (B) Representative flow plots (top) and quantitation of total numbers and phenotypes (bottom) of gp33-specific WT and *Tcf7*^Δ+22kb^ CD8^+^ T cells in LCMV-Arm immune mice 5 days after rechallenge with LCMV-c13. *n* = 12 per genotype, pooled from five experiments, shown with medians and *p* values determined by Mann-Whitney tests. (C) Ratio of WT and *Tcf7*^Δ+22kb^ gp33-specific CD8^+^ T cells following primary LCMV-Arm infection of mixed bone marrow chimeras. *n* = 23, pooled from three experiments. Data analyzed by the paired Wilcoxon test of the ratio of *Tcf7*^Δ+22kb^:WT gp33-specific cells on 8 and 70 dpi LCMV-Arm. (D) Ratio of WT and *Tcf7*^Δ+22kb^ gp33-specific CD8^+^ T cells in LCMV-Arm immune mixed bone marrow chimeras following LM-GP rechallenge. *n* = 18, pooled from two experiments. Data analyzed by the paired Wilcoxon test of the ratio of *Tcf7*^Δ+22kb^:WT gp33-specific CD8^+^ cells pre-rechallenge and 30 dpi with LM-GP. (E) Experimental schematic (top) of competitive adoptive transfer of WT/*Cd19* RNP and *Tcf7*^Δ+22kb^/*Tcf7*^+17kb^ RNP MPEC and SLEC. Log_2_ transformation of the ratio of *Tcf7*^Δ+17kb/+22kb^ to WT P14 cells (bottom left) in sorted MPEC and SLEC from P14 secondary recipients on 21 days post-transfer. Data shown as median and quartiles and were quantified with one-sample *t* and Wilcoxon tests with a theoretical mean of 0 (*n* = 9 SLEC and 5 MPEC from three experiments). Quantitation (bottom middle) and representative histograms (bottom right) of CFSE dilution in MPEC and SLEC on 21 days post-transfer. Data analyzed by two-way ANOVA and uncorrected Fisher’s LSD. (*n* = 4 SLEC and 4 MPEC from two experiments). (F) Experimental schematic (top) of adoptive transfer of secondary WT and *Tcf7*^Δ+22kb^ KLRG1^+^ and KLRG1^−^ memory cells. Quantitation of fold expansion (bottom left) of transferred MPEC and SLEC cells from WT and *Tcf7*^Δ+22kb^ donor mice on 7 dpi LCMV-Arm (*n* = 10 per group from two experiments). Data are shown as medians and were analyzed by two-way ANOVA and uncorrected Fisher’s LSD. (G) TCF-1 and KLRG1 expression in co-transferred WT and *Tcf7*^ΔBlimp1−22^ P14 cells on 8 dpi with LCMV-Arm. *n* = 3–5, representative of two experiments per genotype. Frequencies of indicated populations in each recipient mouse were analyzed by paired *t* test. (H) Analysis of CpG methylation at a *Tcf7*-DMR in CD25^+^ and CD25^−^ P14 cells from WT and *Tcf7*^ΔBlimp1−22^ P14 cells on 5 dpi with LCMV-Arm. Data shown are technical triplicates of *n* = 3 mice and representative of two experiments, analyzed by a Wilcoxon test. (I) Frequencies of tdTomato labeling in total gp33-specific CD8^+^ and TCF-1^+^ KLRG1^+^ and TCF-1^+^ KLRG1^−^ gp33-specific CD8^+^ T cells in *Prdm1*^creERT2/+^ *R26*^tdTomato^ mice after TAM administration on 3 and 4 dpi and analyzed on 6, 10, 21, and 36–40 dpi. Represented as mean ± SEM. See also Figures S4 and S5.

Given that the magnitude of the primary CD8^+^ T cell response was partially attenuated in *Tcf7*^Δ+22kb^ mice, we asked whether retention of TCF-1 in the effector phase impacted memory populations. By 60 dpi with LCMV-Arm, over 90% of gp33-specific CD8^+^ T cells in WT mice expressed TCF-1, and almost all *Tcf7*^*Δ+*22kb^ memory cells were TCF-1^+^ (Figure 5A). KLRG1 expression, which marks effector memory or long-lived effector cells,^36,37^ and CD62L expression, which marks central memory cells,^38^ were comparable between genotypes. Neither total memory nor CD62L^+^ memory T cell numbers differed between genotypes. Since *Tcf7*-deficient CD8^+^ T cells have a deficit in secondary expansion,^14^ we tested whether the retention of *Tcf7* enhances recall expansion. LCMV-Arm-immune WT and *Tcf7*^*Δ*+22kb^ mice were rechallenged with LCMV-c13 on 35 dpi and analyzed 5 days later (Figure 5B). Total numbers of gp33-specific CD8^+^ T cells did not differ between genotypes, although TCF-1 was more highly expressed in *Tcf7*^*Δ+*22kb^ CD8^+^ T cells.

We hypothesized that the lack of an accentuated memory response in *Tcf7*^*Δ+*22kb^ mice was attributable to limitations in cell-extrinsic factors. To assess whether deficiency of the *Tcf7*^+22kb^ conferred cell-intrinsic advantages in memory potential, we reconstituted irradiated mice with a mixture of hematopoietic progenitor cells from congenically distinct WT and *Tcf7*^*Δ+*22kb^ donors and monitored the ratio of WT to *Tcf7*^*Δ+*22kb^ gp33-specific CD8^+^ T cells following LCMV-Arm infection (Figure 5C). The ratio of gp33-specific WT and *Tcf7*^*Δ+*22kb^ CD8^+^ T cells was stable between days 8 and 70 post-infection and was maintained following rechallenge with LM-GP (Figure 5D). Thus, de-repression of *Tcf7* does not enhance memory formation or participation in recall responses. These data are consistent with the report that TCF-1 p45 transgenic mice do not exhibit enhanced anamnestic responses to *Listeria*.^39^

One limitation of these analyses was that we were unable to determine whether KLRG1^+^ CD8^+^ T cells had enhanced memory potential in *Tcf7*^*Δ+*22kb^ mice. To address this, WT and *Tcf7*^Δ+17kb/+22kb^ P14 cells were sorted into KLRG1^hi^ and KLRG1^lo^ populations on 10 dpi, and WT and *Tcf7*^Δ+17kb/+22kb^ cells for each population were mixed at a 1:1 ratio, labeled with Carboxyfluorescein Succinimidyl Ester (CFSE), and adoptively transferred into naive CD45.1 recipient mice (Figure 5E). The ratio of *Tcf7*^Δ+17kb/+22kb^ to WT P14 cells declined by 21 days post-transfer in both transferred populations, indicating that *Tcf7*^Δ+17kb/+22kb^ P14 cells are at a slight disadvantage with respect to memory phase maintenance. Since we did not observe differences in CFSE dilution between genotypes, defects in homeostatic proliferation are likely to be minor.

To address whether *Tcf7*^Δ+22kb^ KLRG1^+^ cells exhibited an advantage in recall responses, we transferred KLRG1^hi^ and KLRG1^lo^ secondary memory cells from WT and *Tcf7*^Δ+22kb^ donors to naive CD45.1 recipients and infected recipient mice with LCMV-Arm on day 21 post-transfer (Figure 5F). WT and *Tcf7*^Δ+22kb^ donor-derived cells expanded equivalently by 7 dpi, indicating that de-repression of *Tcf7* in the effector phase of the response does not promote the maintenance or recall capacity of differentiated effector cells.

### Blimp1 mediates direct but reversible TCF-1 silencing in acute infection

Although disrupting *Tcf7* downregulation during effector differentiation did not influence memory fate specification, we reasoned that identifying upstream regulators of *Tcf7* could provide insight into pathways that restrict memory formation. To determine which factors predominantly mediate TCF-1 downregulation during acute infection, a mixture of congenically distinct WT and *Tcf7*^*ΔAICE−22*^, *Tcf7*^*ΔEbox−22*^, or *Tcf7*^*ΔBlimp1−22*^ P14 cells was adoptively transferred to naive recipients, and their spleens were analyzed 8 dpi with LCMV-Arm (Figure 5G). *Tcf7*^*ΔBlimp1−22*^ P14 cells exhibited the greatest increase in TCF-1 retention relative to internal control cells. *Tcf7*^*ΔAICE−22*^ and *Tcf7*^*ΔEbox−22*^ P14 cells exhibited comparatively minor increases in TCF-1 expression compared with controls (Figures S4A and S4B). These results are consistent with the phenotypes of each TF deficiency (Figures S4C and S4D). *Prdm1^FF^ Cd8-cre* gp33-specific CD8^+^ T cells exhibited a substantial impairment in TCF-1 downregulation on 8 dpi with LCMV-Arm (Figure 3H), whereas only mild to no effects of TFAP4, IRF4, or BATF deficiency were observed on TCF-1 expression at this time point. These results implicate Blimp1 as the major factor directly involved in TCF-1 downregulation *in vivo*.

To gain further mechanistic insight into the role of Blimp1 in repression of *Tcf7*, we profiled how deficiency in the *Tcf7*^+22kb^ Blimp1 binding site impacts *de novo* methylation of *Tcf7*, which has been implicated in its silencing.^40^ Targeted DNA methylation analysis of a previously reported differentially methylated region (DMR) of the *Tcf7* locus^40^ in CD25^+^ and CD25^−^ P14 cells from WT and *Tcf7*^*ΔBlimp1−22*^ on 5 dpi with LCMV-Arm revealed a reduction of methylation in *Tcf7*^*ΔBlimp1−22*^ cells (Figure 5H). The direct binding of Blimp1 to the *Tcf7*^+22kb^ element thus promotes *de novo* methylation during the early phases of antiviral responses, indicating a potential mechanism for the downregulation of *Tcf7*. Our data thus far suggest that upregulation of Blimp1 acts as a critical checkpoint to curtail memory differentiation by repressing *Tcf7* in addition to other essential memory genes. However, it is unclear whether expression of Blimp1 is sufficient for commitment to a short-lived TCF-1^−^ effector state. To assess the impact of early Blimp1 induction on effector fate commitment, *Prdm1^creERT2/+^ Rosa26^LSL-tdTomato/+^* mice were infected with LCMV-Arm and given tamoxifen (TAM) on 3 and 4 dpi, labeling cells that upregulate *Prdm1*. We hypothesized that if Blimp1 enforces commitment of cells to a short-lived effector phenotype, the frequency of fate-mapped cells would decline over time in serially sampled peripheral blood (Figure 5I). Contrary to the hypothesized result, the tdT^+^ cell frequencies continued to increase between 10 and 21 dpi and became constant there-after. Although tdT^+^ cells were initially almost absent among TCF-1^+^ cells, we observed that the frequency of fate mapping in TCF-1^+^ cells increased to 9.02% ± 3.39% by 21 dpi. This increase could be attributed to either superior survival or continued labeling of Prdm1 fate-mapped cells between 6 and 10 dpi. To investigate continuous labeling by residual TAM, we isolated *Prdm1*^*creERT2/+*^ *Rosa26*^*LSL-tdTomato/+*^ P14 on 6 dpi with LCMV-Arm and transferred them into infection-matched secondary recipient mice that had received TAM on 3 and 4 dpi or on 6 dpi (Figure S5A). Under these conditions, we detected similar labeling efficiencies in hosts treated with TAM on 3 and 4 dpi and in hosts treated with TAM at the time of transfer (Figures S5B and S5C). Thus, residual TAM activity contributed to the increase in tdT^+^ cell frequency between 6 and 10 dpi. Nevertheless, *Prdm1* induction does not exclude CD8^+^ T cells from persisting as memory or retaining or regaining TCF-1 expression.

To interrogate *Tcf7* re-activation between 6 and 10 dpi, we used a diphtheria toxin receptor (DTR) transgenic strain of mice, *Tcf7*^*DTR-GFP*^, to deplete *Tcf7*-expressing P14 cells on 4–5 dpi and assess replenishment of *Tcf7*-GFP^+^ cells by 10 dpi (Figure S5D).^13^ Depletion was confirmed in peripheral blood on 6 dpi, with an average of 2.9% P14 cells expressing *Tcf7*^*DTR-GFP*^ in diphtheria toxin (DT)-untreated mice and 0.05% in DT-treated mice (Figure S5E). The frequency of *Tcf7*^*DTR-GFP+*^ P14 cells was approximately 10-fold lower in the spleens of DT-treated mice compared with control mice on 10 dpi (0.291% ± 0.18% vs. 2.43% ± 0.89%, *p* < 0.0001), although this represented a 6-fold increase in *Tcf7*-GFP^+^ frequency relative to 6 dpi (Figure S5F). Moreover, despite efficient ablation on 6 dpi in DT-treated mice, 36.4% ± 9.9% of cells expressed TCF-1 protein by 10 dpi, albeit the majority of these were GFP^−^. These data are consistent with reports that *Tcf7*^hi^ CD8^+^ T cells predominantly give rise to MPECs that maintain high levels of *Tcf7*-GFP transcripts and contribute to central memory.^41^ However, the recovery of cells that express TCF-1 at a protein level provides evidence that *Tcf7* may be reversibly downregulated in a proportion of effector memory-biased cells.^41^ Combined with fate mapping data, these experiments suggest that in a fraction of effector cells, Blimp1 neither permanently silences TCF-1 expression nor curtails persistence in the memory phase.

### *Tcf7*^+22kb^ promotes *Tcf7* silencing during chronic antigen exposure

**Figure 6.**
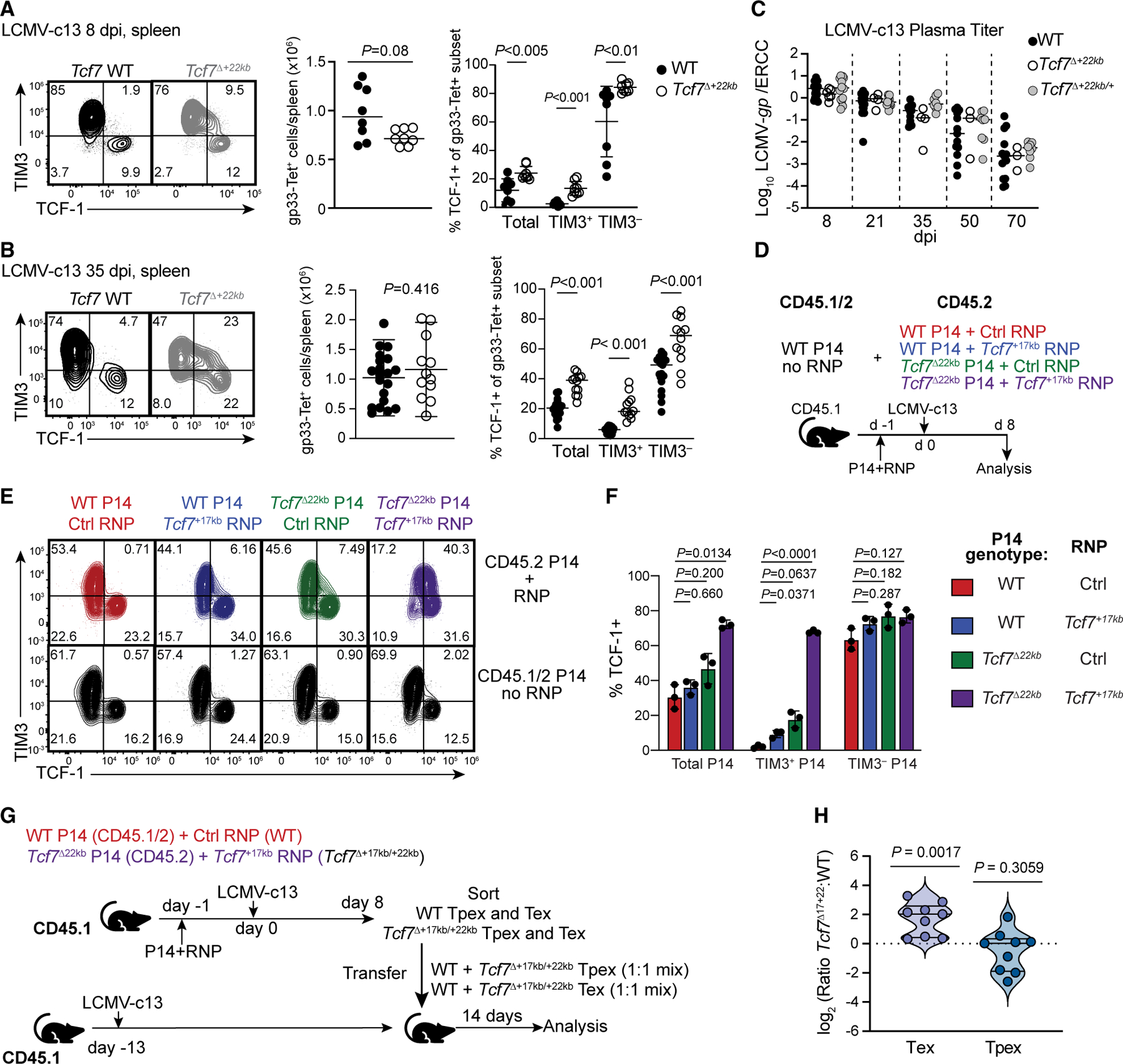
*Tcf7*^+22kb^ mediates TCF-1 downregulation in response to chronic antigen (A) Representative flow plots (left) and quantitation (right) of gp33-specific CD8^+^ T cells from WT and *Tcf7*^Δ+22kb^ mice on 8 dpi with LCMV-c13. Numbers (represented as median, analyzed by Mann-Whitney test) and frequencies (represented as mean ± SD, analyzed by Welch’s *t* test) of each population of gp33-specific CD8^+^ T cells. Pooled from two experiments, *n* = 8. (B) Representative flow plots (left) and quantitation (right) of gp33-specific CD8^+^ T cells from WT and *Tcf7*^Δ+22kb^ mice on 35 dpi with LCMV-c13. *n* = 21 WT and 12 *Tcf7*^Δ+22kb^ mice, pooled from three experiments. Numbers represented as mean ± SD, analyzed by *t* test. Frequencies represented as median, analyzed by Mann-Whitney tests. (C) RT-qPCR analysis of plasma LCMV gp transcripts in LCMV-c13 infected WT, heterozygous, or deficient in *Tcf7*^Δ+22kb^ mice (*n* = 20 for WT, *n* = 6 for *Tcf7*^Δ+22kb/Δ+22kb^, and *n* = 14 for *Tcf7*^Δ+22kb/+^ mice). Data pooled from 4 experiments, shown as medians and analyzed by two-way ANOVA mixed model with Geisser-Greenhouse correction. (D) Experimental scheme for individual and combined perturbation of *Tcf7*^+22kb^ and *Tcf7*^+17kb^ in P14 CD8^+^ T cells. (E) Representative flow plots showing TCF-1 and TIM3 expression in internal control (WT unelectroporated) and P14 cells with indicated RNP/genotype, isolated from the spleen on 8 dpi with LCMV-c13. (F) Frequencies of TCF-1^+^ P14 cells in each experimental group, shown as mean ± SD, and analyzed by two-way ANOVA and Tukey’s multiple comparisons test. *n* = 3. Representative of four experiments. (G) Experimental schematic for adoptive transfer of TIM3^+^ and TIM3^−^ populations of WT/*Cd19* RNP and *Tcf7*^Δ+22kb^/*Tcf7*^+17kb^ RNP P14 cells from 8 dpi LCMV-c13 infected recipients. (H) Log_2_ transformation of the ratio of *Tcf7*^Δ+17kb/+22kb^ to WT P14 cells in sorted TIM3^+^ and TIM3^−^ populations on 14 days post-transfer to P14 secondary recipients. Data shown as median and quartiles and quantified with one-sample *t* and Wilcoxon tests with a theoretical mean of 0 (*n* = 9 per genotype, pooled from two experiments). See also Figure S6.

Next, we determined whether the same regulatory mechanisms applied to persistent antigen responses using LCMV-c13 infection. The percentage of gp33-specific CD8^+^ T cells expressing TCF-1 doubled in *Tcf7*^Δ+22kb^ mice and was twice that of WT mice on 8 dpi in the spleen, blood, and liver (Figures 6A, S6A, and S6B). While TCF-1 expression was barely detected in WT TIM3^+^ CD8^+^ T cells, 13.3% ± 4.9% of TIM3^+^ CD8^+^ T cells expressed TCF-1 in *Tcf7*^Δ+22kb^ mice on 8 dpi, and 21.2% ± 8.0% expressed TCF-1 on 35 dpi (Figure 6B). This was associated with decreased methylation of the *Tcf7* DMR in TIM3^−^ SLAMF6^+^ *Tcf7*^ΔBlimp1−22^ P14 cells on 8 dpi with LCMV-c13 (Figure S6C). Since expression of *Tcf7* from the H2-k^b^ promoter accelerates clearance of LCMV-c13,^15^ we assessed viral titers in plasma of infected mice through 70 dpi in WT, *Tcf7*^Δ+22kb^, and *Tcf7*^Δ+22kb/+^ mice. However, viral titers did not statistically differ from WT mice (Figure 6C).

To determine the effect *of Tcf7*^Δ+22kb^ in the differentiation of tumor-infiltrating lymphocytes (TILs), we inoculated WT:*Tcf7*^Δ+22kb^ mixed bone marrow chimeras with a gp33-expressing MC38 colorectal cancer cell line (MC38.gp). TCF-1^+^ cells in both TIM3^+^ and TIM3^−^ populations were more frequent in *Tcf7*^Δ+22kb^ than in WT TIL (Figures S6D and S6E). Most cells lost expression of TCF-1 in both chronic infection and cancer; therefore, *Tcf7*^+17kb^ may redundantly function in silencing of *Tcf7* expression during antigen persistence.

To test this hypothesis, we electroporated ribonucleoproteins (RNPs) containing Cas9 and sgRNAs targeting the *Tcf7*^+17kb^ element in WT or *Tcf7*^Δ+22kb^ P14 cells, followed by adoptive transfer and LCMV-c13 infection (Figure 6D). Approximately 75% of *Tcf7*^Δ+17kb/+22kb^ cells retained TCF-1 expression, although expression of TCF-1 in TIM3^+^ cells was lower than expression in bona fide Tpex cells (Figures 6E and 6F). Prevention of Blimp1 binding to the *Tcf7* locus did not forestall acquisition of an exhausted phenotype: TIM3 was upregulated normally even when both elements were ablated (Figure 6E). TIM3^+^ cells of both WT and *Tcf7*^Δ+17kb/+22kb^ genotypes downregulated CD73 and SLAMF6, upregulated CX3CR1 and GZMB, and exhibited impaired IFN-γ and tumor necrosis factor (TNF) expression relative to LCMV-Arm effector cells (Figures S6F and S6G). Furthermore, WT and *Tcf7*^Δ+17kb/+22kb^ P14 cells conferred comparable protection to MC38.gp-bearing tumor mice receiving anti-PD-L1 (Figure S6H).

To assess whether retention of TCF-1 conferred *Tcf7*^Δ+17kb/+22kb^ TIM3^+^ cells with increased self-renewal potential, we sorted TIM3^+^ SLAMF6^−^ and TIM3^−^ SLAMF6^+^ WT and *Tcf7*^Δ+17kb/+22kb^ P14 cells on 8 dpi with LCMV-c13, mixed WT and *Tcf7*^Δ+17kb/+22kb^ cells from each population at a 1:1 ratio, and transferred cells to LCMV-c13 infected recipients (Figure 6G). While the ratio of *Tcf7*^Δ+17kb/+22kb^ to WT P14 cells remained constant in the TIM3^−^ SLAMF6^+^ recipients, TIM3^+^ *Tcf7*^Δ+17kb/+22kb^ cells exhibited a competitive advantage over TIM3^+^ WT cells on day 14 post-transfer, indicating that TCF-1 retention imbued TIM3^+^ cells with proliferative capacity (Figure 6H). Therefore, fully breaking the antagonistic circuit between *Prdm1* and *Tcf7* decouples TCF-1 downregulation from terminal exhaustion and promotes the proliferative potential of terminally differentiated cells. However, it does not preserve effector functions or prevent exhaustion of T cells responding to chronic antigen.

### *Prdm1* deficiency rescues differentiation but not secondary expansion of *Tcf7*^−/−^ Tpex

**Figure 7.**
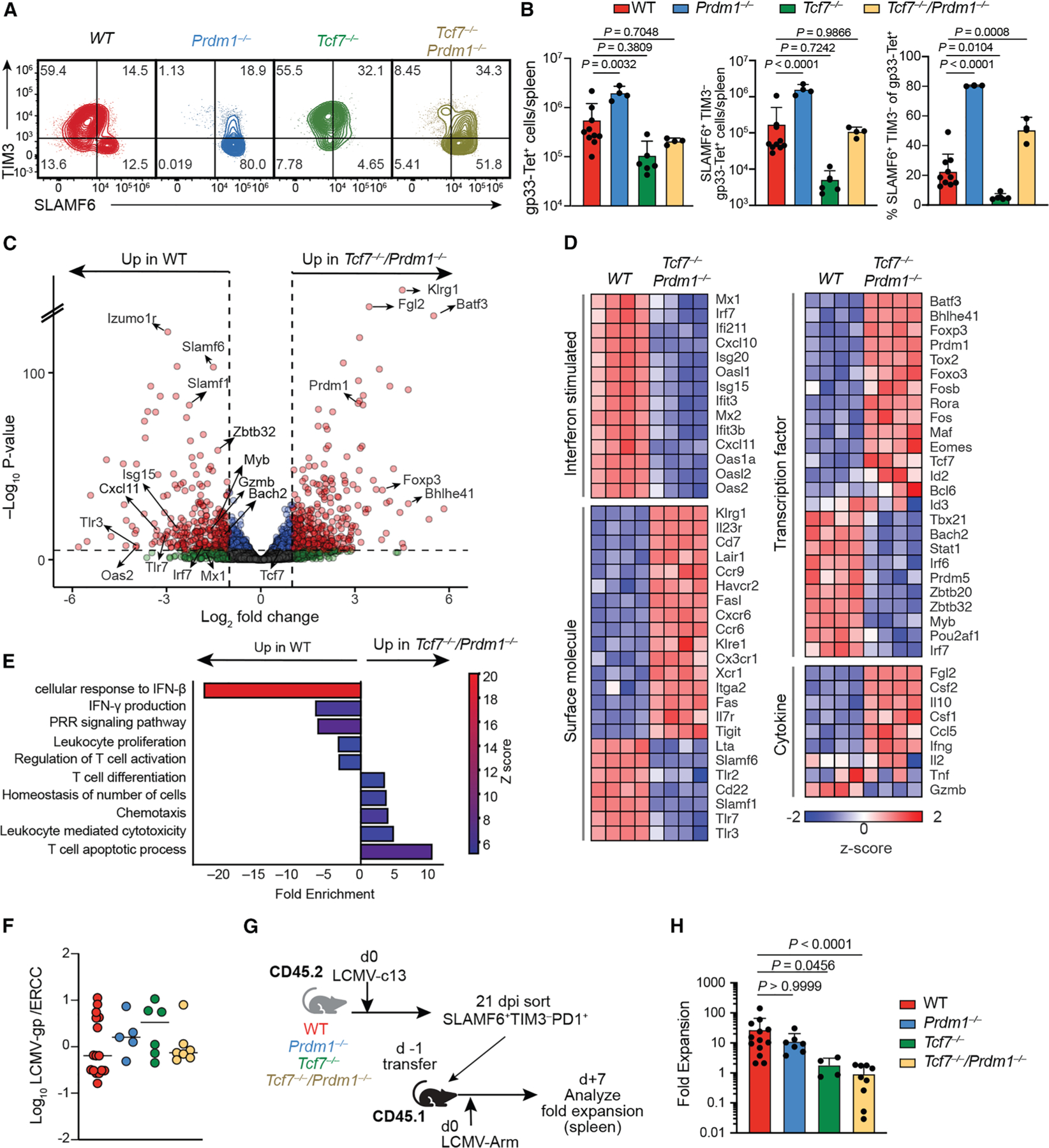
*Prdm1* deficiency rescues the development of *Tcf7*^−/−^ CD8^+^ T cells with the stem-like phenotype but not their recall potential (A) Representative flow plots showing expression of SLAMF6 and TIM3 in WT, *Tcf7*^−/−^, *Prdm1*^−/−^, and *Tcf7*^−/−^*/Prdm1*^−/−^ gp33-specific CD8^+^ T cells from spleens of LCMV-c13 infected mice on 21 dpi (*n* = 9 for WT, *n* = 3 for *Prdm1*^−/−^, *n* = 4 for *Tcf7*^−/−^, and *n* = 3 for *Tcf7*^−/−^*/Prdm1*^−/−^ mice). (B) Pooled data from two experiments shown as mean ± SD and *p* values analyzed by one-way ANOVA and Dunnett’s multiple comparisons test (*n* = 9 for WT, *n* = 3 for *Prdm1*^−/−^, *n* = 4 for *Tcf7*^−/−^, and *n* = 3 for *Tcf7*^−/−^*Prdm1*^−/−^ mice). (C) Volcano plot of RNA-seq results from WT and *Tcf7*^−/−^*/Prdm1*^−/−^ Tpex (*n* = 4) with cutoffs by adjusted *p* < 0.0001 and fold-change > 2. (D) Heatmaps of selected genes of WT and *Tcf7*^−/−^*/Prdm1*^−/−^ Tpex isolated 21 dpi with LCMV-c13 (*n* = 4). (E) GO analysis of differentially expressed genes. (F) Plasma LCMV *gp* transcript abundance in LCMV-c13 infected WT, *Tcf7*^−/−^, *Prdm1*^−/−^, and *Tcf7*^−/−^*/Prdm1*^−/−^ mice (*n* = 16 for WT, *n* = 5 for *Prdm1*^−/−^, *n* = 6 for *Tcf7*^−/−^, and *n* = 7 for *Tcf7*^−/−^*/Prdm1*^−/−^ mice). Data from three experiments, shown as medians and analyzed by one-way ANOVA. (G) Schematic representation of transfer experiment to test recall responses of Tpex, pertaining to (H). (H) Fold-expansion of donor-derived (CD45.2) gp33-specific CD8^+^ T cells of each genotype 7 days after rechallenge, pooled from three experiments (*n* = 13 for WT, *n* = 7 for *Prdm1*^−/−^, *n* = 4 for *Tcf7*^−/−^, and *n* = 9 for *Tcf7*^−/−^*/Prdm1*^−/−^), shown as mean ± SD and analyzed by one-way ANOVA and Dunnett’s multiple comparisons test. See also Figure S7.

Abrogating Blimp1-dependent silencing of *Tcf7* did not skew CD8^+^ T cell differentiation to a Tpex phenotype. We hypothesized that Blimp1 drives terminal differentiation of CD8^+^ T cells independently of *Tcf7* antagonism. We therefore generated *Prdm1*^F/F^ *Tcf7*^F/F^ *Cd8*-cre mice. In mice lacking either *Prdm1* or *Tcf7*, gp33-specific CD8^+^ T cells were respectively skewed toward SLAMF6^+^ TIM3^−^ Tpex-like cells or SLAMF6^−^ TIM3^+^ phenotypes compared with WT mice 21 dpi with LCMV-c13 (Figure 7A). *Tcf7*^−/−^*/Prdm1*^−/−^ CD8^+^ T cells predominantly acquired a SLAMF6^+^ TIM3^−^ Tpex phenotype (Figure 7B). Tpex were reduced 30-fold in *Tcf7*-deficient relative to WT mice. This defect was alleviated in *Tcf7*^−/−^*/Prdm1*^−/−^ animals. Therefore, curtailing effector differentiation by ablating Blimp1 expression nullified the requirement for TCF-1 in maintaining a Tpex surface phenotype.

To address whether the SLAMF6^+^ TIM3^−^ populations in WT and *Tcf7*^−/−^*/Prdm1*^−/−^ mice were phenotypically distinct, we conducted bulk RNA-seq on PD-1^+^ SLAMF6^+^ TIM3^−^ Tpex-like cells from WT and *Tcf7*^−/−^*/Prdm1*^−/−^ mice on 21 dpi with LCMV-c13. We found that 457 genes were downregulated, and 502 genes were upregulated by more than 2-fold in *Tcf7*^−/−^*/Prdm1*^−/−^ relative to WT cells (Figures 7C and 7D). *Tcf7*^−/−^*/Prdm1*^−/−^ cells were enriched for effector-associated genes, including *Klrg1*, *Cx3cr1*, *Fasl*, *Bhlhe41*, and *Ifng*, despite exhibiting the SLAMF6^+^ TIM3^−^ surface phenotype associated with Tpex. Furthermore, several TFs implicated in memory T cell differentiation, including *Bach2*, *Zbtb32*, and *Myb*, were downregulated in *Tcf7*^−/−^*/Prdm1*^−/−^ T cells. Additionally, ISGs were downregulated in *Tcf7*^−/−^*/Prdm1*^−/−^ T cells, as validated by a decrease in IFN-β responses in Gene Ontology (GO) analysis (Figure 7E). The changes in ISG expression were unlikely due to differences in viral burden, as viral titers were comparable between WT, *Prdm1*^−/−^, *Tcf7*^−/−^, and *Tcf7*^−/−^*/Prdm1*^−/−^ mice (Figure 7F). Since a previous report demonstrated that TCF-1 limits IFN-driven T cell exhaustion,^18^ these ISGs are more likely induced by Blimp1 than TCF-1.

To evaluate how counter-regulation of Blimp1 and TCF-1 impacts epigenetic landscapes, we conducted ATAC-seq of SLAMF6^+^ TIM3^−^ PD-1^+^ CD8^+^ cells from WT, *Prdm1*^−/−^, and *Tcf7*^−/−^*/Prdm1*^−/−^ mice 21 dpi with LCMV-c13 (Figure S7A). Comparison of DARs revealed 1,908 peaks that were more accessible in WT cells and 5 peaks that were more accessible in *Prdm1*^−/−^ cells. By contrast, only 32 regions showed decreased accessibility in *Tcf7*^−/−^*/Prdm1*^−/−^ relative to WT cells, and 10 regions exhibited enhanced accessibility in *Tcf7*^−/−^*/Prdm1*^−/−^ cells. ERG, RUNX, and FOS motifs were decreased in *Prdm1*-deficient Tpex, while TCF7L2 motifs were the most enriched motifs among DARs in WT and *Tcf7*^−/−^*/Prdm1*^−/−^ cells (Figures S7B and S7C). While *Prdm1*-deficient Tpex exhibited enrichment of GO terms related to lymphocyte differentiation, T cell signaling, and cytokine responses, *Tcf7*^−/−^*/Prdm1*^−/−^ Tpex exhibited no enrichment of GO annotations (Figure S7D). Thus, *Tcf7* is critical for changes in the chromatin landscape conferred by *Prdm1* deficiency.

To determine whether the *Tcf7*^−/−^*/Prdm1*^−/−^ CD8^+^ T cells with a Tpex phenotype retained proliferative capacity, SLAMF6^+^ TIM3^−^ PD-1^+^ CD8^+^ T cells were purified from WT, *Prdm1*^−/−^, *Tcf7*^−/−^, and *Tcf7*^−/−^*/Prdm1*^−/−^ mice on 21 dpi and were transferred to naive recipients. Their expansion was assessed 7 days after reinfection with LCMV-Arm (Figure 7G). In contrast to WT and *Prdm1*^−/−^ Tpex, which exhibited 20- to 30-fold expansion upon rechallenge, expansion of SLAMF6^+^ TIM3^−^ cells decreased by >10-fold, similar to *Tcf7*^−/−^ Tpex (Figure 7H). *Tcf7*^−/−^*/Prdm1*^−/−^ memory cells from 30 dpi with LCMV-Arm also expanded 6-fold less than WT controls (Figures S7E and S7F). Therefore, even when effector differentiation is restrained in the context of *Prdm1* deficiency, *Tcf7* remains an integral regulator of recall capacity of memory-like cells.

## DISCUSSION

Here, we identified Blimp1 as the major repressor of *Tcf7* in CD8^+^ T cells in both acute and chronic infections. The mode by which Blimp1 represses *Tcf7* is context-specific, as *Tcf7*^+22kb^ and *Tcf7*^+17kb^ both contributed to repression in the presence of antigen prior to LCMV-Arm clearance, in response to IL-12, or during persistent infection with LCMV-c13. Conversely, deletion of *Tcf7*^+22kb^ was sufficient for *Tcf7* de-repression following resolution of acute infection. The mechanisms by which Blimp1 recruitment facilitates *Tcf7* silencing remain incompletely resolved. The reduced DNA methylation of the *Tcf7* DMR in *Tcf7*^ΔBlimp1−22^ P14 cells may play a role in this process, as DNMT3A can be directly recruited to the *Tcf7* locus.^42^ However, this is unlikely to be the dominant mechanism, since a substantial fraction of *Tcf7*^ΔBlimp1−22^ cells undergo methylation at the *Tcf7* DMR. G9A and HDAC2 are recruited to Blimp1 target sites in CD8^+^ T cells, and these factors may additionally repress *Tcf7*.^43^

Notably, even in WT mice infected with LCMV-Arm, a larger proportion of antigen-reactive CD8^+^ T cells expressed TCF-1 on 8 dpi compared with 5 dpi. It is likely that this restoration results from a combination of the selection of cells that did not completely lose TCF-1 and re-activation of *Tcf7* following Blimp1 downregulation, supported by the emergence on 10–21 dpi of TCF-1^+^ cells that were fate mapped by *Prdm1*^creERT2^. Indeed, long-term memory CD8^+^ T cells can derive from cells that previously expressed KLRG1.^44^ Adoptively transferred *Tcf7* reporter^lo^ cells can restore TCF-1 expression over 2 weeks.^45^ Our data indicate that the reversibility of *Tcf7* repression during acute infection is limited, but not necessarily prevented, by *Tcf7*^+22kb^ and *Tcf7*^+17kb^.

Mutagenesis of Blimp1 binding sites in the *Tcf7* locus did not redirect effector cells to a memory or Tpex fate, although subtle defects were noted in primary expansion and effector gene expression of *Tcf7*^+22kb^ T cells in LCMV-Arm. Conversely, de-repression of *Tcf7* supported the expansion capacity of differentiated TIM3^+^ cells in chronic infection, suggesting that TCF-1 functions to preserve proliferative potential. These data affirm the association of TCF-1 with self-renewal of T cells^15,46^ and are reinforced by our finding that *Tcf7*^−/−^*/Prdm1*^−/−^ CD8^+^ T cells fail to expand following rechallenge. In contrast to findings that TCF-1 could curtail effector differentiation in mice overexpressing the p45 isoform of TCF-1, retention of endogenous TCF-1 levels was insufficient to enhance memory.^15,47^ Since TCF-1 can suppress *Prdm1* expression, the de-repression of TCF-1 post-Blimp1 induction may not act early enough to preclude effector differentiation.^21^ While further work will be needed to fully unravel the intricacies of TCF-1 and Blimp1 gene dosage in T cell fate bifurcation, our work suggests that in the context of exhaustion, Blimp1 acts both to enforce the loss of proliferative capacity and the acquisition of an exhausted phenotype. These dual roles of Blimp1 differ in that the former is partially secondary to its direct targeting of TCF-1, while the latter occurs independently of TCF-1 repression.

Collectively, our data indicate that the bifurcation of memory and effector fate, although tightly associated with a transition from a TCF-1^+^ to a Blimp1^+^ state, cannot be fully explained by the retention or loss of TCF-1. Although TCF-1 is obligatory for the repression of some effector-related genes and for recall potential, its retention in Blimp1-replete T cells is permissive for many aspects of effector differentiation. Thus, acquisition of Blimp1 rather than the subsequent loss of TCF-1 may represent the critical checkpoint in effector fate commitment.

### Limitations of the study

Some limitations of the study include the uncertainty regarding the level of *Prdm1* expression required to induce fate mapping, making it challenging to correlate a particular threshold of Prdm1 expression with effector commitment. Therefore, the precise impact of Blimp1 and TCF-1 dosage on T cell fate decisions remains undefined. Additionally, incomplete depletion of TCF-1 protein-expressing cells in the *Tcf7*-DTR experiments confounds interpretation of the data showing partial recovery of TCF-1^+^ cells between 6 and 10 dpi with LCMV-Arm. Thus, the specific cues that instruct T cell differentiation during this critical window of differentiation will need to be more thoroughly resolved in future investigations.

## RESOURCE AVAILABILITY

### Lead contact

Requests for further information and resources should be directed to and will be fulfilled by the lead contact, Takeshi Egawa (egawat@wustl.edu).

### Materials availability

All unique/stable reagents generated in this study are available from the lead contact with a completed materials transfer agreement. We may require a payment and/or a completed materials transfer agreement if there is potential for commercial application.

## STAR★METHODS

### Mice

*Tcf7*^Δ+22kb^ mice were generated by electroporation of C57BL/6N embryos with Cas9/gRNA complexes by the Washington University in St. Louis Department of Pathology and Immunobiology Transgenic Mouse Core. For generation of *Tcf7*^*ΔAICE−22*^, *Tcf7*^*ΔEbox−22*^, and *Tcf7*^*ΔBlimp1−22*^ mouse strains, C57BL/6J embryos were electroporated with Cas9/gRNA RNP complexes and single-stranded oligo-deoxynucleotide homology-directed repair templates. Pups were screened by PCR using Tcf7_+22_F and Tcf7_+22_R primers (for *Tcf7*^+22kb^ deletion), which was followed by BamHI, EcoRI, or NotI digestion for *Tcf7*^*ΔAICE−22*^, *Tcf7*^*ΔEbox−22*^, or *Tcf7*^*ΔBlimp1−22*^ mutations, respectively. All guide, HDR, and primer sequences are contained in Table S1. *Prdm1-cre*^*ERT2*^ mice were generated by embryonic stem cell targeting of the JM8.N4 line. A *Prdm1*-IRES-iCreERT2 cassette was inserted into the 3^′^ untranslated region of *Prdm1*. Correctly targeted clones were injected into *B6(Cg)-Tyr*^*c−2J*^ blastocysts. The resultant chimeras were crossed to *Actb*-Flpe transgenic mice^52^ (JAX) to obtain germline transmission and excise the neomycin resistance cassette. C57BL/6NJ, *Prdm1-YFP* mice,^53^ Batf^−/−^ mice,^54^ *Prdm1*^*F/F*^ mice,^55^ *Tcf7*^*GFP*^ mice,^56^ and *Rosa26*^*LSL-tdTomato*^ (Ai9) mice^57^ were purchased from Jackson Laboratories. B6-CD45.1 mice were purchased from Charles River. The *Tcf7*-flox allele was generated by crossing the *Tcf7*^GFP^ allele to the *Actb*-Flpe transgene. *Irf4* ^*−/−*^ mice^58^ were obtained from Dr. Kenneth Murphy. P14-TCR transgenic mice,^59^ *Tcf7*^*GFP-DTR*^ mice,^50^ *Cd8 (E8I)-cre* mice,^60^ and *Tfap4*^*F/F*^ mice^25^ were previously published.

All experiments were conducted in accordance with animal protocols approved by the Institutional Animal Care and Use Committee (IACUC) at Washington University in St. Louis. Mice used for experiments were aged between 8 and 18 weeks, and cohorts were sex- and age- matched whenever possible. Except where otherwise indicated as *Tcf7*^Δ+22kb/+^, all *Tcf7*^Δ+22kb^ mice used in the study were homozygous for the mutated allele. Likewise, all results reported for *Tcf7*^*ΔAICE−22*^, *Tcf7*^*ΔEbox−22*^, or

*Tcf7^ΔBlimp1−22^* strains were obtained from homozygous mutant cells.

### METHOD DETAILS

#### LCMV Infections

Viruses were propagated in BHK cells and titered by plaque assay using Vero cells, as previously described.^61^ For LCMV-Armstrong infections, mice were injected intraperitoneally with 2 × 10^5^ PFU of virus; for LCMV-c13 infections, mice were injected intravenously with 2 × 10^6^ PFU of virus. Plasma titers of LCMV-c13 were obtained by quantitative PCR of viral RNA in plasma serially sampled from infected mice; titers were normalized to an ERCC spike-in control (Thermofisher, Cat# 4456740), as previously described.^62^ Occasionally, mice did not develop a detectable LCMV tetramer-specific response by

5 dpi with LCMV-Armstrong due to suboptimal infection. These mice were excluded from analysis. The following primer sequences were used: ERCC: 5’-GCTATCAGCTTGCGCCTATTAT-3’ and 5’- GTTGAGTCCACGGGATAGAGTC-3’; LCMV-GP: 5’- CATTCACCTGGACTTTGTCAGACTC-3’ and 5’-GCAACTGCTGTGTTCCCGAAAC-3.’

#### *Listeria monocytogenes* Infections

LM-GP^63^ was purchased from DMX (Cat# DMX 09-080) and glycerol stocks were stored at −80°C. Prior to inoculation, a stock was incubated in 2 mL BHI media for 2 to 4 hours, until reaching an OD_600_ of 0.2 to 0.8. CFU values were estimated by multiplying OD600 by 10^9^. For primary *Listeria* infections, mice were inoculated intravenously with 10,000 CFU of LM-GP. For rechallenge experiments, mice were injected intravenously with 100,000 CFU of LM-GP.

#### Tumor transplantation

MC38-GP cells^64^ were obtained from Dr. Arlene Sharpe. Mice were inoculated subcutaneously with 2–5 × 10^5^ cells resuspended in PBS. Tumor tissue was prepared by mincing the tumors in BSA-free RPMI followed by digestion in 250 μg/mL Liberase (Sigma Aldrich, Cat# 5401020001) and 30 U/mL DNaseI (Sigma Aldrich, Cat# 260913–10MU) at 37°C for 30 min. Digested tumor tissue was resuspended in 4 mL of 40% Percoll; 80% Percoll (Sigma Aldrich, Cat# P1644) was underlaid, and leukocytes were separated following centrifugation.

#### Cas9-sgRNA electroporation

CD8^+^ cells were isolated from WT P14 or *Tcf7*^Δ+22kb^ P14 mice using the MojoSort CD8 T cell negative enrichment kit (Biolegend, Cat# 480035) according to the manufacturer protocol and washed twice in PBS. Cell pellets were resuspended to a density of 10^6^ to 10^8^ cells per 20 μL of P3 Primary Cell Nucleofector Solution pre-mixed at a 4.5:1 ratio with Supplement 1 (Lonza, Cat# V4XP-3032). 1 μL Alt-R Cas9 Electroporation Enhancer was added to the cell suspension prior to addition of RNP complexes. RNP complexes were prepared by mixing 150 pmol sgRNA (synthesized by Synthego) with 61 pmol Alt-R S.p. Cas9 V3 nuclease (IDT, Cat# 1081059) and incubated for 10 minutes at room temperature. For *Tcf7*^+17kb^ targeting, a cocktail of five individually complexed Cas9 RNPs was utilized; RNPs were pooled after complexing (see Table S1). For control electroporations, Cas9 was complexed with either a *Pdcd1*-targeting control guide that did not impact PD-1 expression or a *Cd19* promoter-targeting control guide. A total volume of 5 to 9 μL of RNP mixture was combined with 21 μL of P14 cells and incubated for 2 minutes at room temperature prior to transfer to 16-well Nucleocuvette Strips (Lonza, Cat# V4XP-3032). Cells were electroporated using the 4D Nucleofector pulse setting DN100 (Lonza, Cat# AAF-1003B). A 130 μL volume of 37°C T cell media was added to each well following electroporation; cells were rested in the nucleocuvette strips for 15 minutes at 37°C. T cells were transferred dropwise into three wells of a 96 well U-bottomed plate, and wells were rinsed in an additional 150 μL of 37°C T cell media. Cells were rested for 2 hours at 37°C and subsequently washed once in PBS prior to adoptive transfer to congenic recipient mice.

#### Adoptive transfer experiments

For naive P14 adoptive transfer experiments, P14 cells were either isolated using the MojoSort CD8 T cell negative enrichment kit (Biolegend, Cat# 480035) according to the manufacturer protocol or obtained by bleeding P14 TCR transgenic mice and quantifying P14 cells using precision counting beads (Biolegend, Cat# 424902). Congenically distinct recipients were infected with LCMV one day following adoptive transfer. Recipient mice received 1 × 10^6^ P14 cells for analysis on 3.5 dpi, 1 × 10^5^ P14 cells for analysis on 5 dpi with LCMV-Arm analysis, and 1 × 10^4^ P14 cells for analyses on 8 dpi with LCMV-Arm or LCMV-c13 analysis. For transfers of memory T cells following primary LCMV-Arm infection or LCMV-c13 rechallenge, 5 to 10 × 10^5^ sort-purified KLRG1^+^ CD44^+^ CD8^+^ cells or KLRG1^–^ CD44^+^ CD8^+^ cells were intravenously injected to congenically distinct recipients. For LCMV-c13 Tpex and Tex transfers, 1 to 5 × 10^4^ sort-purified SLAMF6^+^ TIM3^–^ PD-1^+^ CD8^+^ T cells or SLAMF6^–^ TIM3^+^ PD-1^+^ CD8^+^ were transferred to congenically distinct recipients. For memory maintenance experiments, cells were labeled with 10 μM CFSE (Sigma Aldrich 21888 dissolved in DMSO, then diluted in PBS), quenched in FBS, and washed in PBS with 2 mM EDTA and 0.1% BSA prior to transfer. For recall experiments, recipients were challenged with LCMV-Arm one day-post transfer.

For tumor adoptive cellular therapy experiments, P14 cells were isolated using the MojoSort CD8 T cell negative enrichment kit (Biolegend, Cat# 480035) according to the manufacturer’s protocol. Cells were electroporated with either *Cd19* promoter control or *Tcf7*^+17kb^ targeting sgRNAs and activated for 3–4 days with plate-bound anti-CD3/CD28 antibodies. Activated P14 cells were transferred to MC38.GP tumor bearing mice on 10 days post-inoculation with 500,000 tumor cells. 200 μg of anti-PD-L1 (Leinco, Clone 10F.9G2, Cat# P363) were administered on 10-, 13-, and 16-days post inoculation.

#### Diphtheria toxin-mediated depletion

*Tcf7^GFP-DTR^* P14 cells were adoptively transferred to congenic recipient mice one day prior to infection of recipients with LCMV-Arm. 500 ng of Diphtheria toxin (Sigma, Cat# D0564) were injected intraperitoneally on 4 and 5 dpi with LCMV-Arm.

#### Fate mapping

*Prdm1-cre^ERT2^ Rosa26^LSL-tdTomato^* mice were orally gavaged with 10 mg of Tamoxifen (Sigma, Cat# T5648) dissolved in corn oil on 3 and 4 dpi with LCMV-Arm. Mice were bled serially to determine labeling frequency over time.

#### Mixed bone marrow chimeras

One day prior to bone marrow transfer, recipient mice were lethally irradiated with 10.5 Gy. Mice were reconstituted with a 1:1 mixture of WT to *Tcf7*^Δ+22kb^ bone marrow cells; 1 to 5 × 10^7^ bone marrow cells were transferred to each recipient. Recipients were rested for 8 to 16 weeks prior to either LCMV infection or tumor inoculation.

#### *In vitro* CD8^+^ T cell culture

CD8^+^ T cells were isolated according to manufacturer protocols using either the MojoSort CD8 T cell negative enrichment kit (Biolegend, Cat# 480035) or the Dynabeads FlowComp Mouse CD8 isolation kit (Thermofisher, Cat# 114–62D). 200,000 cells were stimulated in each well of 24-well plates pre-coated with polyclonal anti-Hamster IgG (MP bio, Cat# 0855397) in the presence of soluble anti-CD3 (0.1 μg/ml, 145–2C11, BioXcell Cat# BE0001–1), anti-CD28 (1 μg/ml, 37.51, BioXcell, Cat# BE0015–1). For the first 72 hours of culture, cells were stimulated with either 100 U/mL recombinant mouse IL-2 (PeproTech, Cat# 212–12), 10 ng/mL recombinant mouse IL-12 (R&D, Cat# 419-ML-010), or 5 μg/mL anti-IL-2 (S4B6, BioXcell, Cat# BE0043–1, or JES6–1A12, Biolegend, Cat# 503706). Following 72 hours, cells were harvested for staining or passaged into media containing 100 U/mL recombinant mouse IL-2 (PeproTech, Cat# 212–12) or 10 ng/mL recombinant mouse IL-15 (PeproTech, Cat# 200–15) for an additional 72 hours.

#### *Ex vivo* peptide restimulation

Spleens were processed by dissociation between frosted slides in RPMI with 10% FBS. Cells were resuspended in 1 μg/mL of LCMV-gp33–41 (GenScript) and 5 μg/mL Brefeldin A (Biolegend, Cat# 420601) in RPMI with 10% FBS and stimulated for 6 hours prior to staining for flow cytometry.

#### Flow cytometry

Spleens were processed by dissociation between frosted glass slides and filtration through 80 μM nylon filters. Livers were manually dissociated with syringes and filtered through cell strainers. Prior to staining, cells were resuspended in 4 mL of 40% Percoll; 80% Percoll (Sigma Aldrich, Cat# P1644) was underlaid, and leukocytes were separated following centrifugation. PBMCs were separated from EDTA-treated peripheral blood using Lymphopure separation medium according to manufacturer specifications (Biolegend, Cat# 426202). Total viable cell counts were quantified using the Vi-CELL automated cell counter with Trypan blue exclusion (Beckman Coulter). All monoclonal antibodies were purchased from Biolegend, or Thermofisher. Expression of TCF-1 was detected using rabbit anti-TCF-1 monoclonal antibody (Cell Signaling, Clone C63D9, Cat# 2203) and AF488- or AF647- conjugated goat anti-rabbit IgG (AF488: Thermofisher, Cat# R37118; AF647: Biolegend, Cat# 406414). Staining with PE- or APC- labeled LCMV-GP33 and Alexa Fluor (AF) 488-labeled LCMV-NP396 tetramers from the NIH Tetramer Core at Emory was conducted for 30 to 60 minutes at room temperature in PBS with 0.1% BSA and 2.5 mM EDTA. Tetramer was stained concurrently with antibodies targeting cell surface proteins. For in vitro experiments, cells were stained with the viability dyes LIVE/DEAD Aqua (Thermofisher, Cat# L34966) or LIVE/ DEAD Near-IR (Thermofisher, Cat# L34976). For intracellular staining, cells were fixed and permeabilized using the Invitrogen eBioscience Foxp3 / Transcription Factor Staining Buffer kit (Thermofisher, Cat# 50–112-8857). Intracellular staining was performed overnight at 4°C or 30 minutes at room temperature. For samples expressing fluorescent reporter proteins, cells were fixed in 4% paraformaldehyde (Electron Microscopy, Cat# 15710) for 20 minutes at room temperature prior to fixing in the Foxp3/Transcription Factor Staining Buffer kit, and staining for intracellular proteins was performed overnight at 4°C.

Flow cytometry was run using the following instruments: BD FACS LSR Fortessa, X20, or Symphony A3 (BD) or an Aurora 4 Laser V/B/YG/R (Cytek). Cell sorting was performed using a FACSAria-II.

#### RNA isolation and quantitative PCR

Pelleted cells were resuspended in 500 μL TRI Reagent (Sigma, Cat# T9424–200ML). RNA fractions were obtained by phenol/chloroform extraction and isopropanol precipitation. RNA was reverse transcribed using the qScript cDNA SuperMix (Quantabio, Cat# 95048–500). Real time quantitative PCR analysis was performed using Luminaris Color HiGreen qPCR Master Mix (Thermofisher, Cat# K0394) and a LightCycler 480 (Roche). *Tcf7* transcripts were normalized to levels of *Hprt1* housekeeping control transcripts. The following primers were used for qPCR analysis. *Hprt1*: 5’- AGGTTGCAAGCTTGCTGGT-3’ and 5’- TGAAGTACTCATTATAGTCAAGGGCA-3’. *Tcf7*: 5’- TACTCTGCCTTCAATCTGCTCA-3’ and 5’-TGCTGAAATGTTCGTAGAGTGG-3.’

#### ChIP-qPCR

CD8^+^ T cells were stimulated for 48 hours in anti-CD3/CD28 with IL-2 (as described in the *in vitro* T cell culture section) and cross-linked in 1% paraformaldehyde in RPMI for 10 minutes at room temperature prior to quenching with 0.125 M glycine. Cells were washed three times in PBS with 0.1% BSA and 2.5 mM EDTA prior to flash freezing on dry ice. Cells were resuspended in 1 mL of Farnheim lysis buffer (5 mM PIPES pH 8.0, 85 mM KCl, 0.5% NP-40) with protease inhibitor (Sigma, Cat# P8340) and centrifuged at 2000 rpm for 10 minutes to obtain nuclear pellets. Nuclei were resuspended in 1 mL RIPA buffer (1XPBS, 1% NP-40, 0.5% sodium deoxycholate, 0.1% SDS) with protease inhibitor (Sigma, Cat# P8340). RIPA lysates were sonicated on ice for 14 rounds at 60% amplitude, alternating 30 seconds on with 2 minutes off. Sonicated lysates were centrifuged at top speed for 15 minutes at 4°C prior to complexing with antibody-bound beads.

Chromatin immunoprecipitation was performed using the anti-IRF4 antibody (Cell Signaling Technologies, clone D9P5H, 15106S). Per ChIP, 4 μL of anti-IRF4 were incubated with 25 μL of Protein G Dynabeads (Fisher, Cat# 10004D) in 46 μL of 0.5% BSA in PBS for one hour at room temperature. Beads were washed once in 0.5% BSA in PBS prior to combination with lysates. Lysates were incubated with antibody-bound beads overnight at 4°C and washed 5 times in LiCl wash buffer (100 mM Tris pH 7.5, 500 mM LiCl, 1% NP-40, 1% sodium deoxycholate). A final wash was conducted in TE buffer (10 mM Tris-HCl pH 7.5, 0.1 mM EDTA). Chromatin was eluted and reverse-crosslinked in 200 μL of 1% SDS with 0.1 M NaHCO_3_ overnight at 65°C and cleaned up with the QIAquick PCR Purification Kit (Qiagen, Cat# 28104). Purified DNA was quantified by qPCR, as described in the quantitative PCR section. For positive control sequences, the following primer sets were used: Bcor^+65^: 5’- GGGAGGGGCTTACCTCCTCC-3’ and 5’- CACTCACGGAGAGGAACCATTC-3’; Enpp6^–45^: 5’- ATAGCCAGA GCGTGACCTGC-3’ and 5’- CTGCAGGACAGTGATTGGCCG-3’; Tcf7^+22kb^: 5’- GGTTCCACCCAGCCCCAGGG-3’ and 5’- GGT GGTGGTGATGGCTGATTC-3.’

#### Bulk RNA-sequencing

Cell pellets of SLAMF6^+^ TIM3^–^ PD1^+^ CD8^+^ T cells sorted from WT and *Tcf7*^*−/−*^*/Prdm1*^*−/−*^ mice on 21 dpi LCMV-c13 were resuspended in 500 μL TRI Reagent. RNA was purified according to the manufacturer protocol using the Direct-zol RNA Microprep Kit (Zymo Research, Cat# R2062). RNA quality was assessed using an Agilent 2100 Bioanalyzer. Libraries were prepared using the Takara-Clontech SMARTer kit and sequenced for 300 cycles on a NovaSeq X Plus, targeting 30 million reads per library (Illumina). Data were demultiplexed using DRAGEN and BCLconvert version 4.2.4 software and aligned to the Ensembl release 101 primary assembly with STAR version 2.7.9a.^65^ Heat maps were generated using Phantasus and associated packages.^66^ Differentially expressed genes were defined as having an adjusted *P* value <0.05 and a log_2_ Fold change > 1. Volcano plots were generated using the edgeR,^67^ DESeq2,^68^ and EnhancedVolcano packages in R. Gene IDs were mapped to GO terms using org.Mm.eg.db in R, and clusterProfiler was used for gene ontology enrichment analysis. Once a CSV was generated in R, data representation was performed in Python. Plots were generated using Seaborn.

#### Bulk ATAC-sequencing

10,000 to 50,000 freshly sorted cells from LCMV-c13-infected mice on 21–22 dpi were treated with 200 U/mL DNase (Worthington, Cat# LS002007) in RPMI culture medium for 30 minutes at 37°C. Cells were washed twice in cold PBS to remove residual DNase. The lysis and transposition reactions were performed as previously described^69^ using the Illumina Tagment DNA TDE1 Enzyme and Buffer Small Kit (Illumina, Cat# 20034197). Libraries were indexed using a custom-synthesized set of oligonucleotides for 22 dpi Tpex and Tex datasets (Figure 1A), or the IDT for Illumina DNA UD Index Set D (Illumina, Cat# 20027213) for comparison of 21 dpi Tpex from *Tcf7*^*−/−*^*/Prdm1*^*−/−*^ and *Prdm1*^*−/−*^ mice (Figures 7C–7F). Amplified libraries were cleaned up using double-sided bead purification with AMPure XP beads (Beckman Coulter, Cat# A63880). Library quality was assessed by Qubit fluorometric quantification prior to pooling indexed libraries.

Libraries were sequenced for 300 cycles on a NovaSeq X Plus (for the *Tcf7*^*−/−*^*/Prdm1*^*−/−*^ dataset) or on a HiSeq 2500 (for the 22 dpi exhaustion subset dataset), targeting 50 million reads per library (Illumina). For the analysis of Tpex/Tex ATAC-sequencing data in Figure 1A, reads were mapped to the mm9 genome using the package Bowtie2, and HOMER^70^ was used to find peaks and generate bedGraphs of the data. BedGraphs were visualized in the UCSC genome browser. For *Tcf7*^*−/−*^*/Prdm1*^*−/−*^ and *Prdm1*^*−/−*^ Tpex analysis, reads were analyzed using the ENCODE ATACseq pipeline (v2.2.0) and mapped to the mm10 genome. Differential accessibility analysis was performed using the DESeq2 package in R with a log_2_ fold change cutoff of >1.5 and FDR of <0.05. Peaks were annotated with the package GenomicRanges^71^ using the reference GTF file from GENCODE release M24. Differentially accessible motifs were identified using the findMotifsGenome.pl function in HOMER. Volcano plots were generated using the EnhancedVolcano package. Gene ontology analysis was performed using the clusterProfiler package in R.

#### scATAC-seq library preparation, sequencing, and alignment

scATAC-seq experiments were performed on the 10x Chromium platform, following a previously described protocol.^72^ Briefly, after sorting, cells were washed with PBS containing 0.04% BSA and subjected to nuclei isolation as per the manufacturer’s instructions. Nuclei were counted, and approximately 10,000 nuclei were used for tagmentation. The tagmented nuclei were then loaded for capture using the 10x Chromium controller. After gel emulsion generation, linear amplification was performed, followed by DNA purification following the manufacturer’s protocol. The resulting DNA was used for library construction, as described on the manufacturer’s website. The libraries were quantified using an Agilent Bioanalyzer and sequenced on an Illumina NovaSeq S4 sequencer, using the following setup: 50 bp read 1N, 8 bp i7 index, 16 bp i5 index, and 50 bp read 2N. In this reaction, 1N and 2N refer to the DNA insert sequencing, whereas i5 and i7 sequencing identify the individual barcodes of single cells. The resulting reads were aligned with CellRanger to mm10 genome.

#### scATAC-seq data processing and analysis

scATAC analysis was done with ArchR (version 1.0.2) to perform preprocessing, dimensional reduction, and clustering. scATAC-seq were read with the ArchR package and filtered out cell barcodes with less than 100 fragments per cell and a TSS enrichment less than 10. Doublets were identified and filtered with ‘addDoubletScores’ and ‘filterDoublets’ functions. Dimensional reduction was performed using ‘addIterativeLSI’ function with the default parameter. We then perform clustering using ‘addClusters’ function with resolution set to be 1.0. To identify cluster specific peaks and a consensus peakset for the downstream analysis, pseudo-bulk ATAC replicates were created for each cluster, and chromatin accessibility peaks were called using MACS2, implemented in ‘addReproduciblePeakSet’ function. Gene activity scores were calculated with an exponential smoothing kernel with promoter peaks defined as scATAC peaks within 2,000 bp upstream or 100 bp downstream of a TSS. The transcription factors binding site motif enrichment was estimated by chromVAR using ‘addDeviationMatrix’ with the ‘cisbp’ motif annotation database. UMAP visualization was performed with addUMAP and then, plotEmbedding function with default parameters. To assess differential cell abundance between wild-type and *Tcf7*^*Δ+22kb*^ samples, we utilized the miloR R package.^73^ Briefly, we constructed a k-nearest neighbors (KNN) graph from the reduced dimension space of single-cell ATAC-seq, defined representative neighborhoods, and performed differential abundance testing using a negative binomial generalized linear model. The differences were visualized on the UMAP embedding to highlight regions with altered cell populations between conditions.

#### Single-cell RNA-seq library preparation

Single-cell RNA-seq libraries were generated using the 10x Genomics Single Cell Immune Profiling Solution Kit (v2 Chemistry) according to the manufacturer’s instructions. Briefly, cells were isolated by FACS and washed with PBS containing 0.04% BSA. After reverse transcription and barcoding of individual cells within droplets, and cDNA was purified with Dynabeads MyOne SILANE after breaking the emulsion, followed by PCR amplification under the following conditions: 98°C for 45 seconds, 14 cycles of 98°C for 20 seconds, 67°C for 30 seconds, and 72°C for 1 minute, with a final extension at 72°C for 1 minute. For gene expression library construction, 50 ng of amplified cDNA was fragmented, end-repaired, and underwent double-sided size selection using SPRIselect beads, followed by PCR amplification with indexing primers (98°C for 45 seconds, 14 cycles of 98°C for 20 seconds, 54°C for 30 seconds, and 72°C for 20 seconds, with a final extension at 72°C for 1 minute). After another double-sided size selection with SPRIselect beads, libraries were sequenced on an Illumina NovaSeq 6000 to a target depth of at least 25,000 reads per cell, using 28 bp for Read 1, an 8 bp i7 index, and 91 bp for Read 2.

#### scRNA-seq data processing and analysis

Reads from 10X scRNA expression libraries were aligned to mouse genome assembly mm10 and quantified using cellranger count (10x Genomics, v3.1.0). The filtered feature-barcode matrices were used for downstream analysis. Single cell gene expression matrices were imported into the R environment and analyzed using Seurat R Package. Cells with >500 genes captured and >3000 UMIs were included in downstream analyses. Additionally, cells with >6% mitochondria reads were excluded from subsequent analyses. The scRNA-seq libraries from each sample were merged with ‘merge’ function and subsequently, the merged matrix were normalized with ‘NormalizeData’ function and scaled with top 2500 variable gene using ‘FindVariableFeatures’ and then ‘ScaleData’ functions. Dimensional reduction was performed with ‘RunPCA’ with default parameters. UMAP visualization was generated with ‘RunUMAP’. Clusters were defined by constructing a k-nearest neighbors graph and identifying groups of cells with ‘FindClusters’ function with resolution of 0.5. Cell type specific marker genes were nominated with ‘FindAllMarkers’ with logfc.threshold = 0.2.

#### Targeted DNA methylation sequencing/analysis

DNA was isolated from sort-purified P14 CD8^+^ T cells from wild-type and *Tcf7*^ΔBlimp1−22^ donors isolated on 8 dpi and 21 dpi with LCMV-c13 and on 5 dpi of LCMV-Armstrong and bisulfite converted using the EZ DNA Methylation-Direct Kit (Zymo Research, Cat# D5021). Bisulfite-converted genomic DNA was used for PCR amplification for a key differentially methylated region in the *Tcf7* locus using the following primers: mTcf7 (Forward primer: 5’-GGTTAGTTTGAGTTTGGTTTAGAGTAGTGAG-3’, Reverse primer: 5’- CCTCTTACCTAAATTTCCCTACAAAATACC-3’), followed by verification of the amplicon DNA size by gel electrophoresis. The amplified DNA was purified using the Zymoclean Gel DNA Recovery Kit (Zymo Research, Cat# D4008). The purified amplicon DNA was then used for library preparation using the Native Barcoding Kit 24 V14 (Oxford Nanopore Technologies). The barcoded DNA library was sequenced using an R10 flow cell for sequencing on an Oxford Nanopore MinION Mk1B sequencer, as previously described. The generated FASTQ files were processed for genome alignment and subsequent analysis to determine the percentage of CpG methylation of the amplified region DMR using a customized NanoEM pipeline.

#### Statistical Analysis

The software Prism 10 (GraphPad) was used for all statistical analyses. We used two-tailed Student’s t-test, Mann Whitney U-test for unpaired data or Wilcoxon test for two group comparisons, and one way- or two way- ANOVA or the Kruskal-Wallace test for multi-group comparisons with either Tukey’s, Fisher’s Least Significant Difference, Sidak’s, or Dunnett’s post hoc, as indicated in each figure legend.

## Supplementary Material

MMC1

MMC2

SUPPLEMENTAL INFORMATION

Supplemental information can be found online at https://doi.org/10.1016/j.immuni.2025.09.008.

## Figures and Tables

**Table:** KEY RESOURCES TABLE

REAGENT or RESOURCE	SOURCE	IDENTIFIER
Antibodies		

AF488-conjugated anti-rabbit IgG	Thermofisher	Cat#A11034; RRID: AB_2576217
AF647-conjugated anti-rabbit IgG	BioLegend	Cat#406414; RRID: AB_2563202
APC-Cy7-conjugated anti-B220 (Clone:RA3-6B2)	BioLegend	Cat#103224; RRID: AB_313006
BV510-conjugated anti-B220 (Clone:RA3-6B2)	BioLegend	Cat# 103247; RRID: AB_2561394
PerCP-Cy5.5-conjugated anti-B220 (Clone: RA3-6B2)	BioLegend	Cat#103236; RRID: AB_893354
BV605-conjugated anti-B220 (Clone:RA3-6B2)	BioLegend	Cat#103243; RRID: AB_11203907
PE-Dazzle-conjugated anti-B220 (Clone:RA3-6B2)	BioLegend	Cat#103258; RRID: AB_2564053
APC-conjugated anti-CCR7 (Clone:4B12)	BioLegend	Cat#120108; RRID: AB_389233
PE-Cy7-conjugated anti-CD11b (Clone:M1/70)	BioLegend	Cat#101216; RRID: AB_312798
BV421-conjugated anti-CD127 (Clone:A7R34)	BioLegend	Cat#135024; RRID: AB_10897948
AF700-conjugated anti-CD19 (Clone:6D5)	BioLegend	Cat#115528; RRID: AB_493734
APC-conjugated anti-CD25 (Clone:PC61)	BioLegend	Cat#102012; RRID: AB_312860
BV605-conjugated anti-CD25 (Clone:PC61)	BioLegend	Cat#102035; RRID: AB_11126977
FITC-conjugated anti-CD25 (Clone:PC61)	BioLegend	Cat#102006; RRID: AB_312854
PE-conjugated anti-CD25 (Clone:PC61)	BioLegend	Cat#102008; RRID: AB_312856
APC-conjugated anti-CD4 (Clone:GK1.5)	BioLegend	Cat#100412; RRID: AB_312696
APC-Cy7-conjugated anti-CD4 (Clone:GK1.5)	BioLegend	Cat#100414; RRID: AB_312698
BUV395-conjugated anti-CD4 (Clone:GK1.5)	BD	Cat#563790; RRID: AB_2738426
BV510-conjugated anti-CD4 (Clone:RM4-5)	BioLegend	Cat#100559; RRID: AB_2561388
BV711-conjugated anti-CD4 (Clone:RM4-5)	BioLegend	Cat#100550; RRID: AB_2562099
BV785-conjugated anti-CD4 (Clone:RM4-5)	BioLegend	Cat#100551; RRID: AB_2563053
PerCP-Cy5.5-conjugated anti-CD4 (Clone:GK1.5)	BioLegend	Cat#100434; RRID: AB_893324
AF700-conjugated anti-CD44 (Clone:IM7)	BioLegend	Cat#103026; RRID: AB_493712
Pacific Blue-conjugated anti-CD44 (Clone:IM7)	BioLegend	Cat#103020; RRID: AB_493682
AF700-conjugated anti-CD45 (Clone:30-F11)	BioLegend	Cat#103128; RRID: AB_493714
BV570-conjugated anti-CD45.1 (Clone:A20)	BioLegend	Cat#110733; RRID: AB_10895765
BV605-conjugated anti-CD45.1 (Clone:A20)	BioLegend	Cat#110738; RRID: AB_2562565
PE-Cy7-conjugated anti-CD45.1 (Clone:A20)	BioLegend	Cat#110730; RRID: AB_1134170
AF700-conjugated anti-CD45.2 (Clone:104)	BioLegend	Cat#109822; RRID: AB_493730
APC-Cy7-conjugated anti-CD45.2 (Clone:104)	BioLegend	Cat#109824; RRID: AB_830788
FITC-conjugated anti-CD45.2 (Clone:104)	BioLegend	Cat#109806; RRID: AB_313442
APC-Fire750-conjugated anti-CD62L (Clone: MEL-14)	BioLegend	Cat#104450; RRID: AB_2629771
FITC-conjugated anti-CD62L (Clone:MEL-14)	BioLegend	Cat#104406; RRID: AB_313092
PE-conjugated anti-CD62L (Clone:MEL-14)	BD	Cat#553151; RRID: AB_394666
PerCP-Cy5.5-conjugated anti-CD62L (Clone: MEL-14)	BioLegend	Cat#104432; RRID: AB_2187123
FITC-conjugated anti-CD69 (Clone:H1.2F3)	BioLegend	Cat#104506; RRID: AB_313108
APC-Cy7-conjugated anti-CD8a (Clone:53-6.7)	BioLegend	Cat#100714; RRID: AB_312752
BUV395-conjugated anti-CD8a (Clone:53-6.7)	BD	Cat#565968; RRID: AB_2732919
BV785-conjugated anti-CD8a (Clone:53-6.7)	BioLegend	Cat#100749; RRID: AB_11218801
PE-Cy7-conjugated anti-CD8a (Clone:53-6.7)	BioLegend	Cat#100722; RRID: AB_312760
BV605-conjugated anti-CD8a (Clone:53-6.7)	BioLegend	Cat#100744; RRID: AB_2562609
FITC-conjugated anti-CD8a (Clone:53-6.7)	BioLegend	Cat#100706; RRID: AB_312744
PerCP-Cy5.5-conjugated anti-CD8b (Clone: YTS156.7.7)	BioLegend	Cat#126610; RRID: AB_2260149
BV605-conjugated anti-CX3CR1 (Clone: SA011F11)	BioLegend	Cat#149027; RRID: AB_2565937
BV785-conjugated anti-CX3CR1 (Clone: SA011F11)	BioLegend	Cat#149029; RRID: AB_2565938
BV711-conjugated anti-CXCR5 (Clone:L138D7)	BioLegend	Cat#145529; RRID: AB_2734207
PE-Dazzle-conjugated anti-CXCR6 (Clone: SA051D1)	BioLegend	Cat#151117; RRID: AB_2721699
PE-cy5-conjugated anti-Gzmb (Clone:QA16A02)	BioLegend	Cat#372226; RRID: AB_2910421
AF700-conjugated anti-Ki67 (Clone:B56)	BD	Cat#561277; RRID: AB_10611571
BV650-conjugated anti-Ki67 (Clone:B56)	BD	Cat#563757; RRID: AB_2688008
APC-Cy7-conjugated anti-KLRG1 (Clone:2F1)	BioLegend	Cat#138426; RRID: AB_2566553
FITC-conjugated anti-KLRG1 (Clone:2F1)	BioLegend	Cat#138410; RRID: AB_10643582
PE-Cy7-conjugated anti-KLRG1 (Clone:2F1)	BioLegend	Cat#138416; RRID: AB_2561735
PE-Cy7-conjugated anti-LAG3 (Clone:C9B7W)	Thermofisher	Cat#25-2231-82; RRID: AB_2573428
BB700-conjugated anti-Ly108 (Clone:13G3)	BD	Cat#742272; RRID: AB_2871448
BV605-conjugated anti-Ly108 (Clone:13G3)	BD	Cat#745250; RRID: AB_2742834
BV650-conjugated anti-NK1.1 (Clone:PK136)	BioLegend	Cat#108735; RRID: AB_11147949
BV750-conjugated anti-PD1 (Clone:29F.1A12)	BioLegend	Cat#135263; RRID: AB_2941421
FITC-conjugated anti-PD1 (Clone:29F.1A12)	BioLegend	Cat#135214; RRID: AB_10680238
PE-Cy7-conjugated anti-PD1 (Clone:29F.1A12)	BioLegend	Cat#135216; RRID: AB_10689635
PE-Cy7-conjugated anti-Sca1 (Clone:E13-161.7)	BioLegend	Cat#122514; RRID: AB_756198
BV650-conjugated anti-SLAM (Clone:TC15-12F12.2)	BioLegend	Cat#115931; RRID: AB_2562402
PE-Cy7-conjugated anti-Tbet (Clone:4B10)	BioLegend	Cat#644823; RRID: AB_2561760
Unconjugated anti-TCF1 (Clone:C63D9)	Cell Signaling	Cat#2203S; RRID: AB_2199302
APC-Cy7-conjugated anti-TCR Va2 (Clone:B20.1)	BioLegend	Cat#127818; RRID: AB_10682897
FITC-conjugated anti-TCR Vb8.1,8.2 (Clone: KJ16-133.18)	BioLegend	Cat#118406; RRID: AB_1227787
AF700-conjugated anti-Thy1.1 (Clone:OX-7)	BioLegend	Cat#202528; RRID: AB_1626241
Pacific Blue-conjugated anti-Thy1.1 (Clone:OX-7)	BioLegend	Cat#202522; RRID: AB_1595477
PE-conjugated anti-Thy1.1 (Clone:OX-7)	BioLegend	Cat#202524; RRID: AB_1595524
PerCP-Cy5.5-conjugated anti-Thy1.2 (Clone:53-2.1)	BioLegend	Cat#140322; RRID: AB_2562696
BV421-conjugated anti-TIM3 (Clone:RMT3-23)	BioLegend	Cat#119723; RRID: AB_2616908
eFluor660-conjugated anti-TOX (Clone:TXRX10)	Thermofisher	Cat#50-6502-82; RRID: AB_2574265
PerCP/Fire 806 -conjugated anti-LAG3 (Clone:C9B7W )	BioLegend	Cat#125249; RRID: AB_3068219
APC-conjugated anti-TIGIT (Clone:VSIG9)	BioLegend	Cat#142106; RRID: AB_10960139
Unconjugated anti-IRF4 (Clone:D9P5H)	Cell Signaling	Cat#15106S; RRID: AB_2798709
PE-Dazzle-conjugated anti-CD73 (Clone: TY/11.8)	BioLegend	Cat#127233; RRID: AB_2800628
anti-PD-L1(Clone: 10F.9G2)	Leinco	Cat# P363; RRID:AB_2749826
anti-CD3 (Clone: 145-2C11)	BioXcell	Cat # BE0001-1; RRID: AB_1107634
anti-CD28 (Clone: 37.51)	BioXcell	Cat # BE0015-1; RRID: AB_1107624
anti-Hamster IgG	MP bio	Cat#0855397
anti-IL-2 (Clone: S4B6)	BioXcell	Cat # BE0043-1; RRID: AB_1107705
anti-IL-2(Clone: JES6-1A12)	BioLegend	Cat # 503706; RRID: AB_315291

Bacterial and virus strains		

LCMV-Armstrong	prepared in lab	N/A
LCMV-clone 13	prepared in lab	N/A
Listeria monocytogenes-GP	DMX	Cat#09-080

Chemicals, peptides, and recombinant proteins		

Carboxyfluorescein succinimidyl ester (CFSE)	Sigma Aldrich	Cat#21888
LCMV-gp33-41 peptide	GenScript	custom ordered
ERCC Spike-in control RNA	Thermofisher	Cat# 4456740
DNaseI	Sigma Aldrich	Cat # 260913-10MU
Liberase	Sigma Aldrich	Cat#5401020001
Cas9 V3 nuclease	IDT	Cat#1081059
Diphtheria toxin	Sigma Aldrich	Cat # D0564
Tamoxifen	Sigma Aldrich	Cat #T5648
Recombinant mouse IL-2	PeproTech	Cat # 212-12
Recombinant mouse IL-12	R&D	Cat # 419-ML-010
Recombinant mouse IL-15	PeproTech	Cat # 200-15
Brefeldin A	Biolegend	Cat# 420601
4% paraformaldehyde	Electron Microscopy	Cat# 15710
DNase	Worthington	Cat # LS002007

Critical commercial assays		

Alt-R Cas9 Electroporation Enhancer	IDT	Cat#1075915
P3 Primary Cell 4D-Nucleofector^®^ X Kit S	Lonza	Cat# V4XP-3032
Precision Count Beads	BioLegend	Cat # 424902
Dynabeads FlowComp Mouse CD8 isolation kit	Thermofisher	Cat #114-62D
MojoSort CD8 T cell negative enrichment kit	Biolegend	Cat # 480035
Lymphopure lymphocyte separation medium	Biolegend	Cat # 426202
LIVE/DEAD Aqua	Thermofisher	Cat # L34966
LIVE/DEAD Near-IR	Thermofisher	Cat # L34976
eBioscience Foxp3 / Transcription Factor Staining Buffer kit	Thermofisher	Cat # 50-112-8857
TriReagent	Sigma	Cat # T9424-200ML
qScript cDNA SuperMix	Quantabio	Cat # 95048-500
Luminaris Color HiGreen qPCR Master Mix	Thermofisher	Cat # K0394
Protease inhibitor cocktail	Sigma Aldrich	Cat# P8340
QIAquick PCR Purification Kit	Qiagen	Cat# 28104
Direct-zol RNA Microprep Kit	Zymo Research	Cat# R2062
Tagment DNA TDE1 Enzyme and Buffer Small Kit	Illumina	Cat # 20034197
AMPure XP beads	Beckman Coulter	Cat # A63880
EZ DNA Methylation-Direct Kit	Zymo Research	Cat # D5021
Zymoclean Gel DNA Recovery Kit	Zymo Research	Cat # D4008
Native Barcoding Kit 24 V14	Oxford Nanopore Technologies	Cat# SQK-NBD114.24
10x Genomics Single Cell Immune Profiling Solution Kit		N/A

Deposited data		

scATAC-seq of 8 dpi LCMVArm	this paper	GEO: GSE295247
scRNA-seq of 8 dpi LCMVArm	this paper	GEO: GSE295246
Bulk ATAC-seq of TPEX and TEX	this paper	GEO: GSE294878
Bulk ATACseq of WT, Prdm1, Tcf7, and DKO TPEX	this paper	GEO: GSE294879
Bulk RNA-seq of Prdm1 fate mapped effector T cells	this paper	GEO: GSE294880
Bulk RNA-seq of WT, Prdm1, Tcf7, and DKO TPEX	this paper	GEO: GSE294881
Bulk RNA-seq of WT and Tcf7Δ+17kb/+22kb effector T cells	this paper	GEO: GSE294882
Bulk ATAC-seq of naive, SLEC, and Tcm cells	Scott-Browne et al.^48^	GEO: GSE88987
ChIP-seq of AP4 in CD8+ T cells	Chou et al.^25^	GEO: GSE58075
ChIP-seq of BATF and IRF4 in CD4+ T cells	Iwata et al.^49^	GEO: GSE85172
Raw data corresponding to main figures	this paper	Mendeley Data: https://doi.org/10.17632/6xjrfdh9pz.1
Raw data corresponding to supplemental figures	this paper	Mendeley Data: https://doi.org/10.17632/yrn6xk4227.1
ChIP-seq of Blimp1 in CD8+ T cells	Mackay et al.^20^	GEO: GSE79339

Experimental models: Cell lines		

MC38.gpLaboratory of Arlene Sharpe	Laboratory of Arlene Sharpe	N/A

Experimental models: Organisms/strains		

Mouse: *Tcf7*^*Δ+22kb*^	This paper	N/A
Mouse: *Tcf7*^*ΔAICE−22*^	This paper	N/A
Mouse: *Tcf7*^*ΔEbox−22*^	This paper	N/A
Mouse: *Tcf7*^*ΔBlimp1−22*^	This paper	N/A
Mouse: *Prdm1*^*creERT2*^	This paper	N/A
Mouse: C57BL/6-cBrd/cBrd/Cr (C57BL/6 albino)	Charles River	Strain #:562
Mouse: Actb-Flpe	Jackson Laboratory	Strain #:005703; RRID:IMSR_JAX:005703
Mouse: C57BL/6NJ	Jackson Laboratory	Strain #:005304; RRID:IMSR_JAX:005304
Mouse: *Prdm1*^YFP^	Jackson Laboratory	Strain #:008828; RRID:IMSR_JAX:008828
Mouse: Batf^−/−^	Jackson Laboratory	Strain #:013758; RRID:IMSR_JAX:013758
Mouse: *Prdm1*^F/F^	Jackson Laboratory	Strain #:008100; RRID:IMSR_JAX:008100
Mouse: *Tcf7*^GFP^	Jackson Laboratory	Strain #:030909; RRID:IMSR_JAX:030909
Mouse: Rosa26LSL-tdTomato (Ai9)	Jackson Laboratory	RRID:IMSR_JAX:007909
Mouse: Irf4^−/−^	Laboratory of Kenneth Murphy	Strain #:031834; RRID:IMSR_JAX:031834
Mouse: P14-TCR transgenic	Jackson Laboratory	MMRRC Strain #037394-JAX; RRID: MMRRC_037394-JAX
Mouse: *Tcf7*^GFP-DTR^	Siddiqui et al.^50^	N/A
Mouse: Cd8 (E8I)-cre mice	Jackson Laboratory	Strain #:008766; RRID:IMSR_JAX:008766
Mouse: *Tfap4*^F/F^	Chou et al., 2015	N/A
Mouse: B6-CD45.1 congenic	Charles River Laboratories	RRID: IMSR_CRL:564
Mouse: B6-CD45.1 isogenic	Jackson Laboratory	Strain #:033076; RRID:IMSR_JAX:033076

Oligonucleotides		

See Table S1 for oligonucleotide sequences		N/A

Software and algorithms		

Cell Ranger	10x Genomics	
Seurat R Package	Stuart et al.^51^ PMID: 31178118	https://satijalab.org/seurat
ArchR (version 1.0.2)	Granja et al.^35^; PMID: 33633365	RRID: SCR_020982 https://www.nature.com/articles/s41587-019-0332-7
Prism 10	GraphPad	https://www.graphpad.com/; RRID:SCR_002798
FlowJo (version 10.9.0)	Treestar	https://www.flowjo.com/; RRID:SCR_008520

Other		

AF488-conjugated anti-H-2Db FQPQNGQFI	NIH Tetramer core	N/A
PE-conjugated anti-H2-Db KAVYNFATC	NIH Tetramer core	N/A
APC-conjugated anti-H2-Db KAVYNFATC	NIH Tetramer core	N/A

## Data Availability

The raw and processed RNA-seq and ATAC-seq data files are deposited at GEO (GEO: GSE295247, GSE295246, GSE294878, GSE294879, GSE294881, GSE294882). Raw data files are deposited on Mendeley Data (Mendeley Data: https://doi.org/10.17632/6xjrfdh9pz.1, https://doi.org/10.17632/yrn6xk4227.1). Any additional information required to reanalyze data reported in this paper will be available from the lead contact. This paper does not report original code.

## Highlights

- *Tcf7*^+22kb^ is necessary for *Tcf7* silencing at the peak of the acute Teff response
- *Tcf7*^+22kb^ and *Tcf7*^+17kb^ redundantly silence *Tcf7* in chronic or early acute infection
- Blimp1 mediates *Tcf7* silencing but not effector commitment
- TCF-1 preserves proliferative potential of stem-like CD8^+^ T cells
