# Supplementary material for: The transcriptional repressor BLIMP1 enforces TCF-1-dependent and -independent restriction of the memory fate of CD8+ T cells
Source: Immunity. Author manuscript; Available in PMC 2025 Dec 20. (PMC12717841; doi:10.1016/j.immuni.2025.09.008)
Supplement: MMC1 [file NIHMS2115159-supplement-MMC1.pdf]

Immunity, Volume *58*

Supplemental information

The transcriptional repressor BLIMP1 enforces TCF-1-dependent and -independent restriction of the memory fate of CD8$^+$ T cells

Maegan K. Murphy, Matthew McCullen, Joshua L. Deffenbaugh, Andy Y. Chen, Joy Pai, Bence Daniel, Amir Yousif, Saravanan Raju, Sunnie Hsiung, Zhenxiao Wang, Hazem E. Ghoneim, Ansuman T. Satpathy, Marco Colonna, Eugene M. Oltz, and Takeshi Egawa

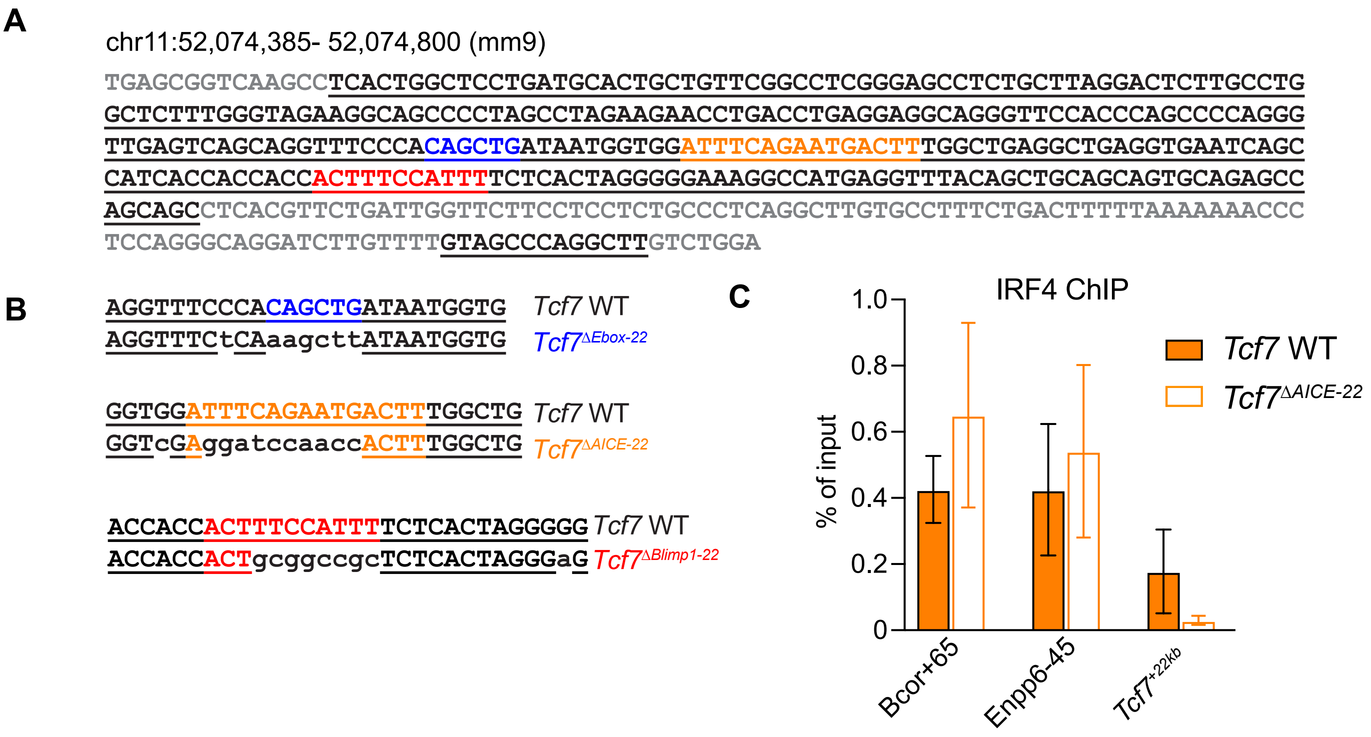

Figure S1. Generation and validation of *Tcf7*$^{\Delta+22kb}$ mice, Related to Figures 1 and 2.
(A) Chromosomal coordinates and sequence of *Tcf7*$^{+22kb}$. The sequence that was deleted by Cas9 mediated cleavage in the germline of *Tcf7*$^{\Delta+22kb}$ mice is underlined. Ebox (AP4 binding site), AICE (IRF4/BATF binding site), and Blimp1 binding motifs are marked in blue, orange, and red, respectively. (B) Sequences of WT (above) and TF binding site mutant (below) mice at each transcription factor binding site. Mutated sequences are in black lowercase text without underlines. (C) ChIP-qPCR analysis to asssess IRF4 binding in WT and *Tcf7*$^{\Delta AICE\text{-}22}$ CD8^{+} T cells activated for 2 days with αCD3/28 + 100 U/mL IL-2, N=2. Data presented as mean ± SD.

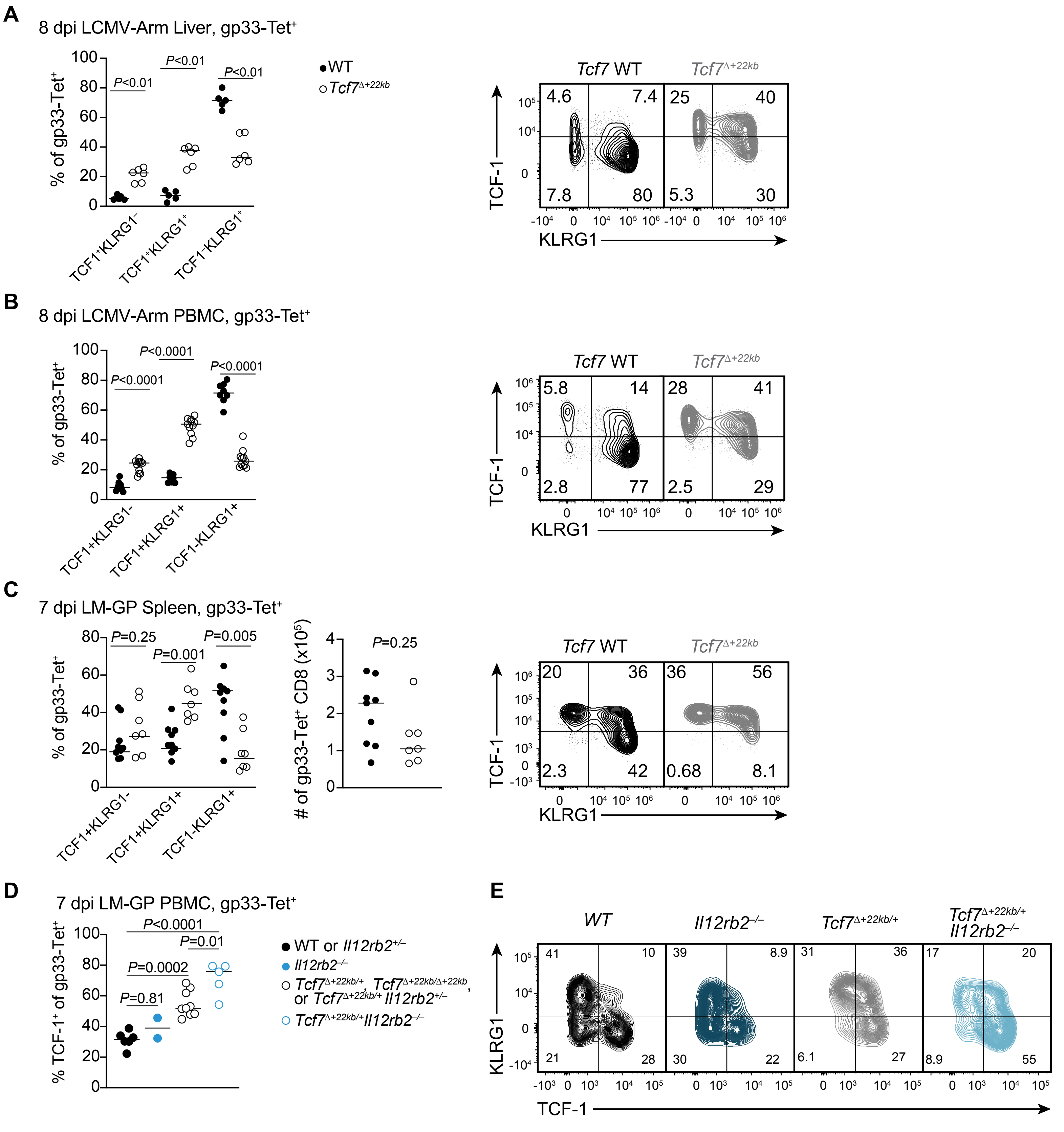

Figure S2. ***Tcf7*$^{+22kb}$ is required for silencing of *Tcf7* in CD8^{+} T cells in multiple tissues following acute LCMV infections, Related to Figure 3.** (A and B) Quantitation and representative flow cytometry plots of gp33-specific WT and *Tcf7*$^{\Delta+22kb}$ CD8^{+} T cells on 8 dpi with LCMV-Arm in the liver (A, n=4 for WT and n=5 for *Tcf7*$^{\Delta+22kb}$ mice) and in PBMC (B, n= 8 for WT and n=11 for *Tcf7*$^{\Delta+22kb}$ mice), pooled from two independent experiments shown with medians, and analyzed by Mann-Whitney tests.
(C) Quantitation and representative flow cytometry plots of gp33-specific WT and *Tcf7*$^{\Delta+22kb}$ CD8^{+} T cells on 7 dpi with LM-GP in the spleen, pooled from two independent experiments, shown as medians, and Analyzed by Mann-Whitney test. n=9 for WT and n=7 for KO mice. (D) Quantification and (E) representative flow plots of frequency TCF-1^{+} of gp33-Tet^{+} CD8^{+} T cells from peripheral blood on 7 dpi with LM-GP. Data were pooled from two independent experiments (n=2 WT, 4 *Il12rb2*$^{+/-}$, 2 *Il12rb2*$^{-/-}$, 5 *Tcf7*$^{\Delta22kb/+}$, 3 *Tcf7*$^{\Delta22kb/+}$ *Il12rb2*$^{+/-}$, 1 *Tcf7*$^{\Delta22kb}$, and 5 *Tcf7*$^{\Delta22kb/+}$ *Il12rb2*$^{-/-}$) and analyzed by one-way ANOVA.

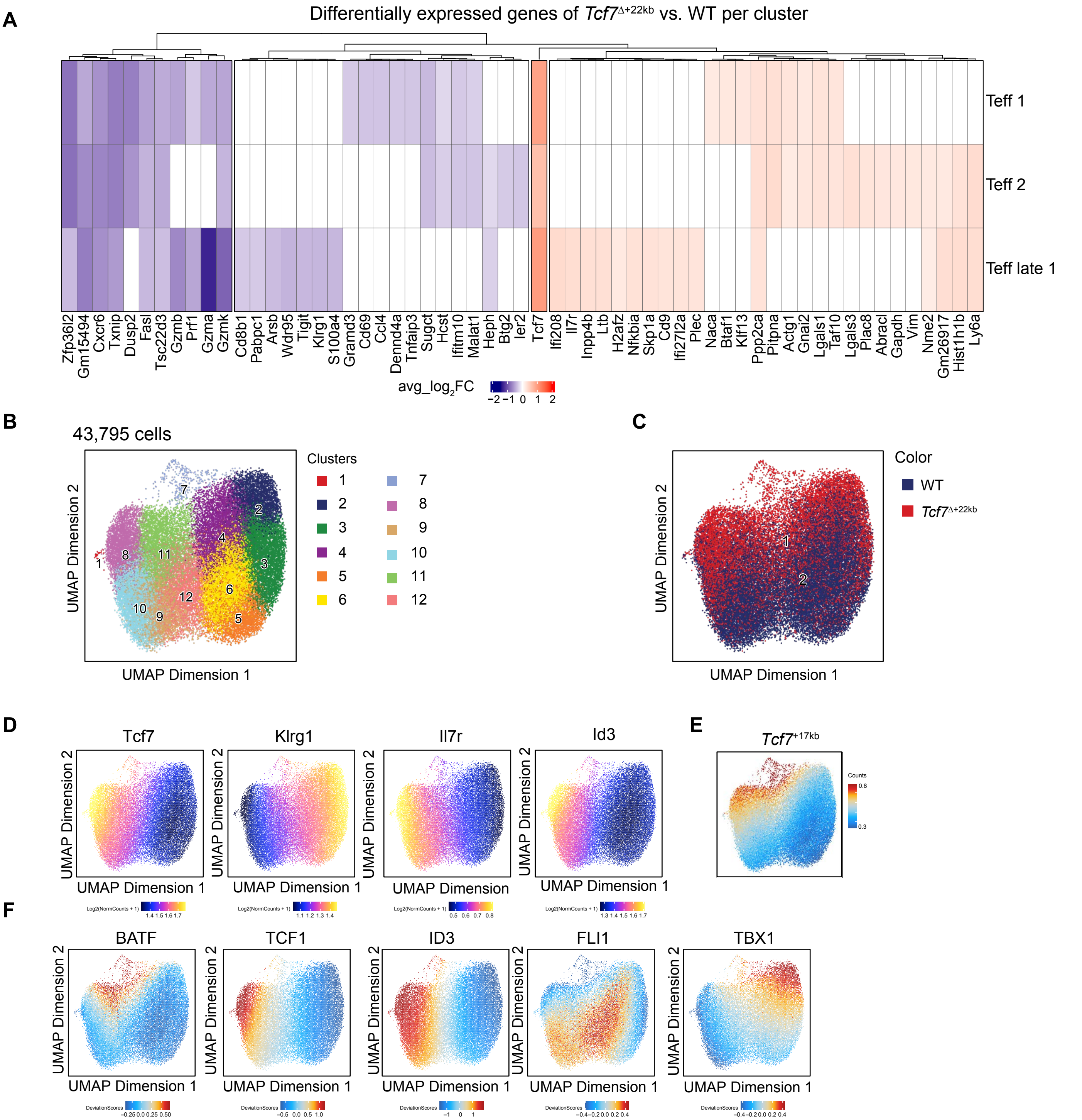

Figure S3. ***Tcf7*** $^{+22kb}$ **regulates the transcriptional and epigenetic states of CD8⁺ T cells in acute LCMV infection, Related to Figure 4.**

(A) Heatmap of differentially expressed genes in WT and *Tcf7* $^{\Delta+22kb}$ cells in scRNA-seq clusters Teff1, Teff2, and Teff late 1.

(B) UMAP projection of scATAC-seq data, comprised of gp33-Tet⁺ CD8⁺ T cells sorted 8 dpi LCMV-Arm.

(C) UMAP colored by genotype (pooled from two mice per genotype).

(D) UMAP visualization of gene activity scores of selected genes.

(E) Feature plot of *Tcf7* $^{+17kb}$ accessibility in gp33-Tet⁺ cell scATAC-seq dataset.

(F) UMAP visualization of the motif deviation scores of selected transcription factors.

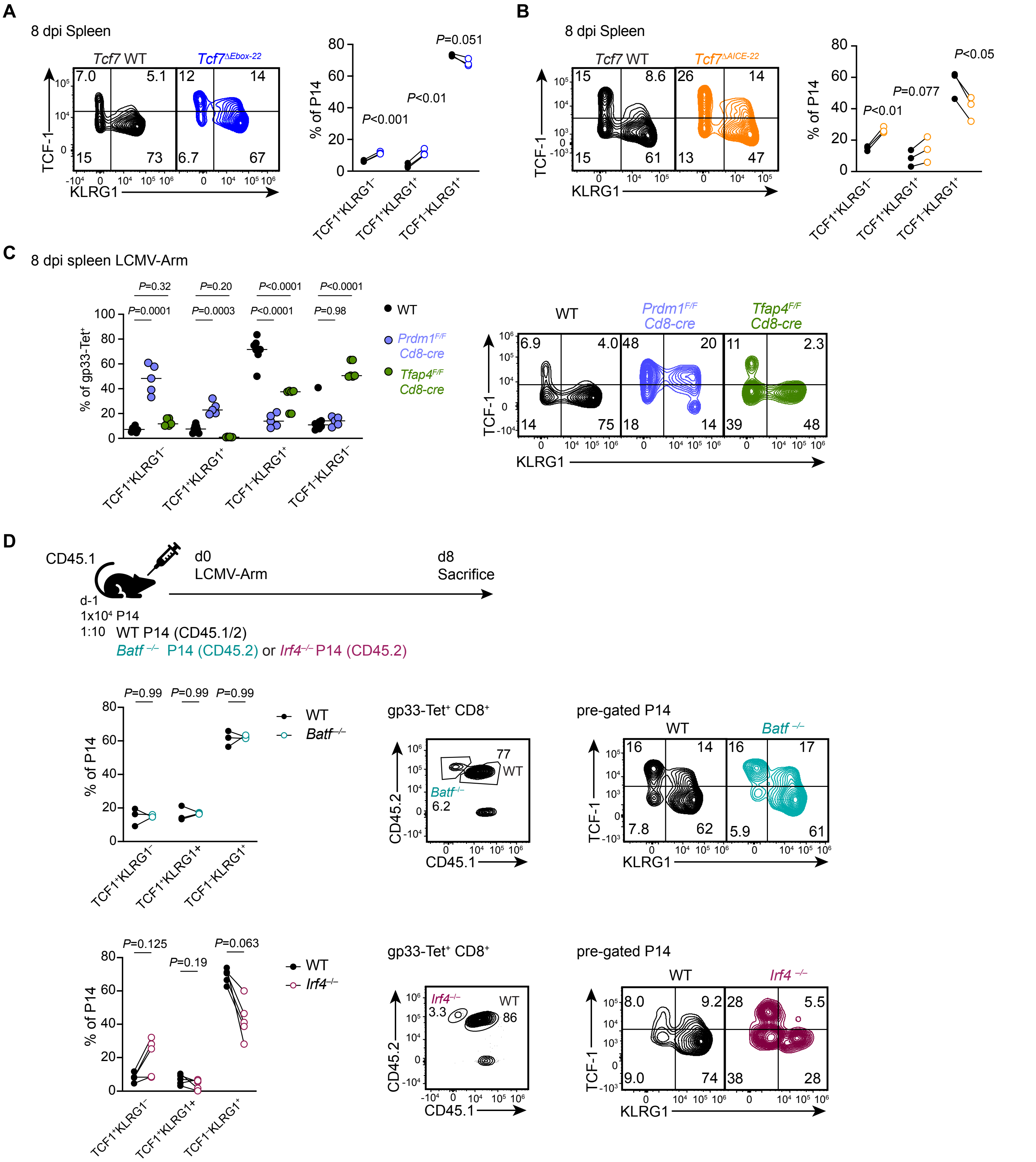

Figure S4. Effects of transcription factor deficiency on TCF-1 expression in $CD8^+$ T cells responding to LCMV-Arm infection, Related to Figure 5. (A and B) TCF-1 and KLRG1 expression in congenically distinct, co-transferred WT and indicated *Tcf7*$^{\Delta Ebox\text{-}22}$ (A) and *Tcf7*$^{\Delta AICE\text{-}22}$ (B) P14 cells on 8 dpi with LCMV-Arm. n=3, representative of two independent experiments per genotype. Frequencies of indicated populations in each recipient mice were analyzed by paired t-test (C) Population frequencies (left) and representative flow cytometry plots (right) of splenic gp33-specific $CD8^+$ T cells from indicated genotypes of mice on 8 dpi with LCMV-Arm infection. Data were analyzed by two-way ANOVA and Tukey's multiple comparison test. Data were pooled from two independent experiments (n=8 WT, 5 *Prdm1*$^{F/F}$ *Cd8-cre*, and 6 *Tfap4*$^{F/F}$ *Cd8-cre* mice).(D) Representative schematic (top) and frequencies and representative flow plots (bottom) of co-transferred WT and *Batf*$^{-/-}$ or *Irf4*$^{-/-}$ P14 cells 8dpi of LCMV-Arm infection of congenic recipients. Data were analyzed by multiple Wilcoxon tests.

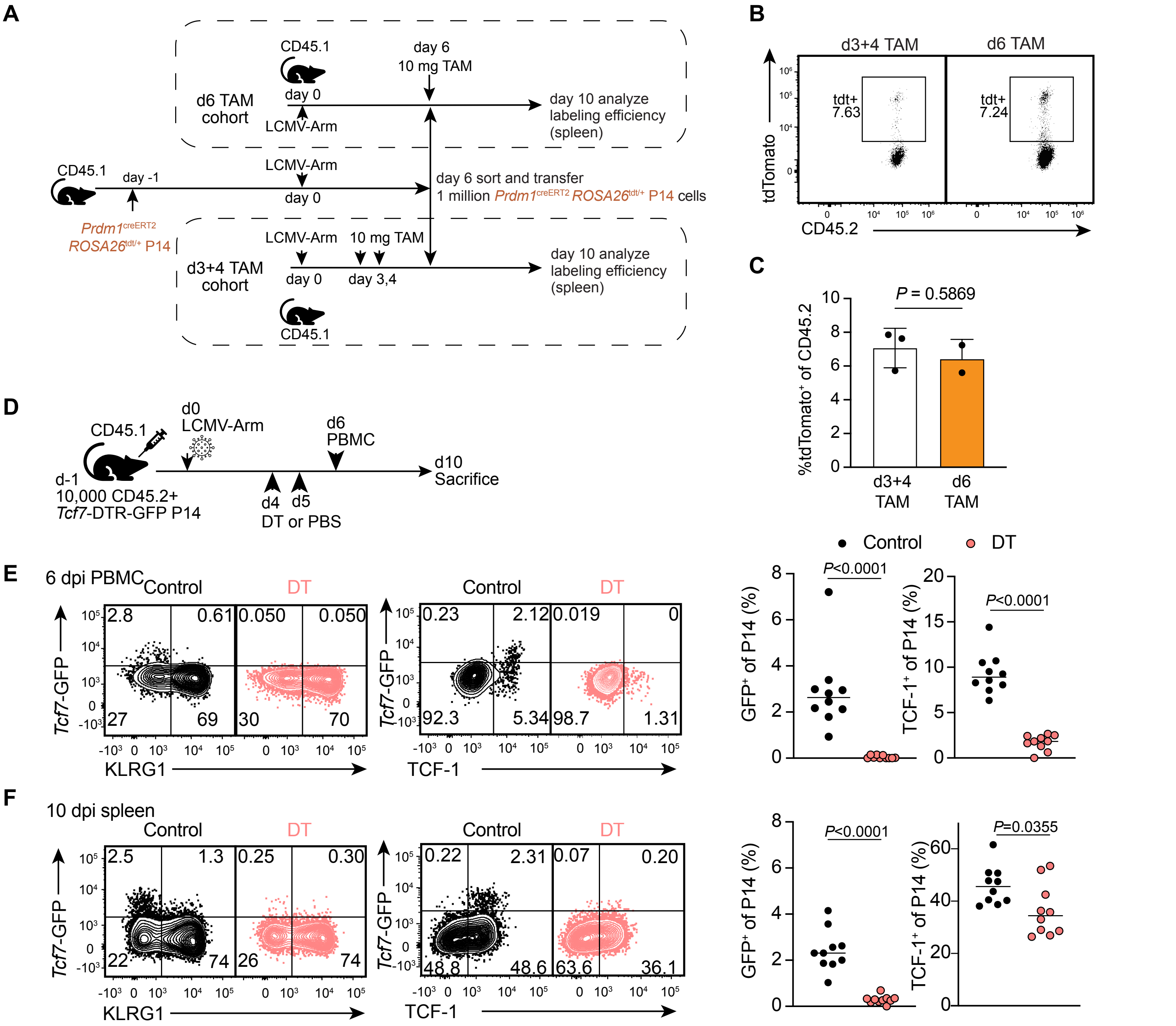

Figure S5. *Prdm1* expression is permissive for memory differentiation in acute LCMV infection, Related to Figure 5. (A) Experimental schematic for panels B and C. (B) Representative flow cytometry data of frequency of fate-mapped P14 cells on 10 dpi. (C) Quantitation of frequency of fate-mapped P14 cells on 10 dpi (n=3 d3+4 TAM recipients and 2 d6 TAM recipients). Data presented as mean±SD and analyzed by t-test. (D) Graphical representation of experimental approach pertaining to panels (E) and (F). (E and F) Representative flow cytometry plots (left) and frequencies (right) of adoptively transferred *Tcf7*$^{DTR\text{-}GFP+}$ P14 cells in PBMC on 6 dpi (E) and in the spleen on 10 dpi (F). n=10 for DT-treated and n=10 for PBS treated recipients. Data pooled from two independent experiments are presented with median and analyzed by Mann-Whitney test.

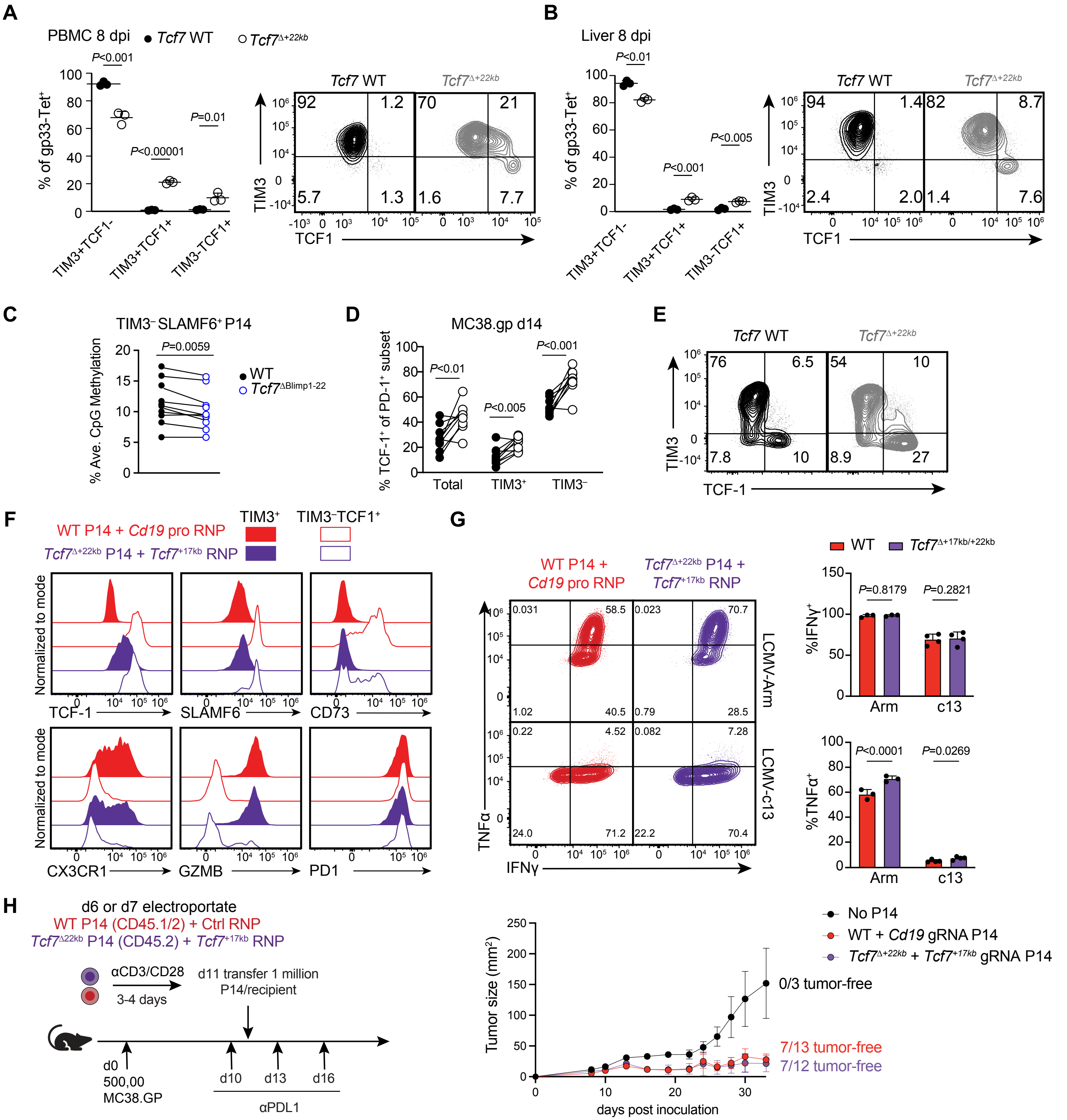

Figure S6. *Tcf7*$^{+22kb}$ deficiency results in retention of TCF-1 expression in CD8$^+$ T cells responding to LCMV-c13 infection or MC38.gp tumor cells, Related to Figure 6. (A,B) Quantitation (left) and representative flow plots (right) of gp33-Tet$^+$ CD8$^+$ T cells in PBMC and livers of WT and *Tcf7*$^{\Delta+22kb}$ mice 8 dpi LCMV-c13. Data are represented as mean±SD and analyzed by t-test. (C) Bisulfite sequencing analysis of mTcf7-DMR percent CpG methylation in TIM3$^-$SLAMF6$^+$ P14 cells from WT and *Tcf7*$^{\Delta Blimp1-22}$ P14 cells on 8 dpi LCMV-c13. Data are technical triplicate of n=3 mice and representative of two independent experiments; data were analyzed by Wilcoxon test. (D) Percentages and (E) representative flow plots of each population of all PD1$^+$ tumor infiltrating lymphocytes of each genotype in *Tcf7*$^{\Delta+22kb}$:WT mixed bone marrow chimeric mice d14 post inoculation with MC38.gp tumor cells (n=9, combined from two independent experiments). Data were analyzed by paired t-tests. (F) Representative histograms of indicated markers of exhausted T cell populations in TIM3$^+$ and TIM3^x cells from *Tcf7*$^{\Delta+22kb}$ + *Tcf7*$^{+17kb}$ RNP P14 to WT + *Cd19* RNP P14 on 8 dpi with LCMV-c13. (G) Representative flow plots (left) and graphical representation (right) of cytokine expression of WT and *Tcf7*$^{\Delta17+22}$ P14 cells 6 hours post-peptide restimulation of *ex vivo* cells from LCMV-Arm or LCMV-c13 infected mice (n=3 LCMV-Arm, 4 LCMV-c13). Data representative of two independent experiments are shown as mean±SD and analyzed by 2-way ANOVA. (H) Experimental approach (left) and tumor growth curves (right) for mice inoculated with MC38.gp tumors and treated with adoptive cell therapy on 10 days post inoculation and anti-PD-L1 on 10, 13, and 16 days post inoculation. Data pooled from three independent experiments were shown as mean±SEM (n=3 mice receiving no P14 cells, 13 WT + *Cd19* gRNA P14 recipients and 12 *Tcf7*$^{\Delta+22kb}$ + *Tcf7*$^{+17kb}$ gRNA P14 recipients).

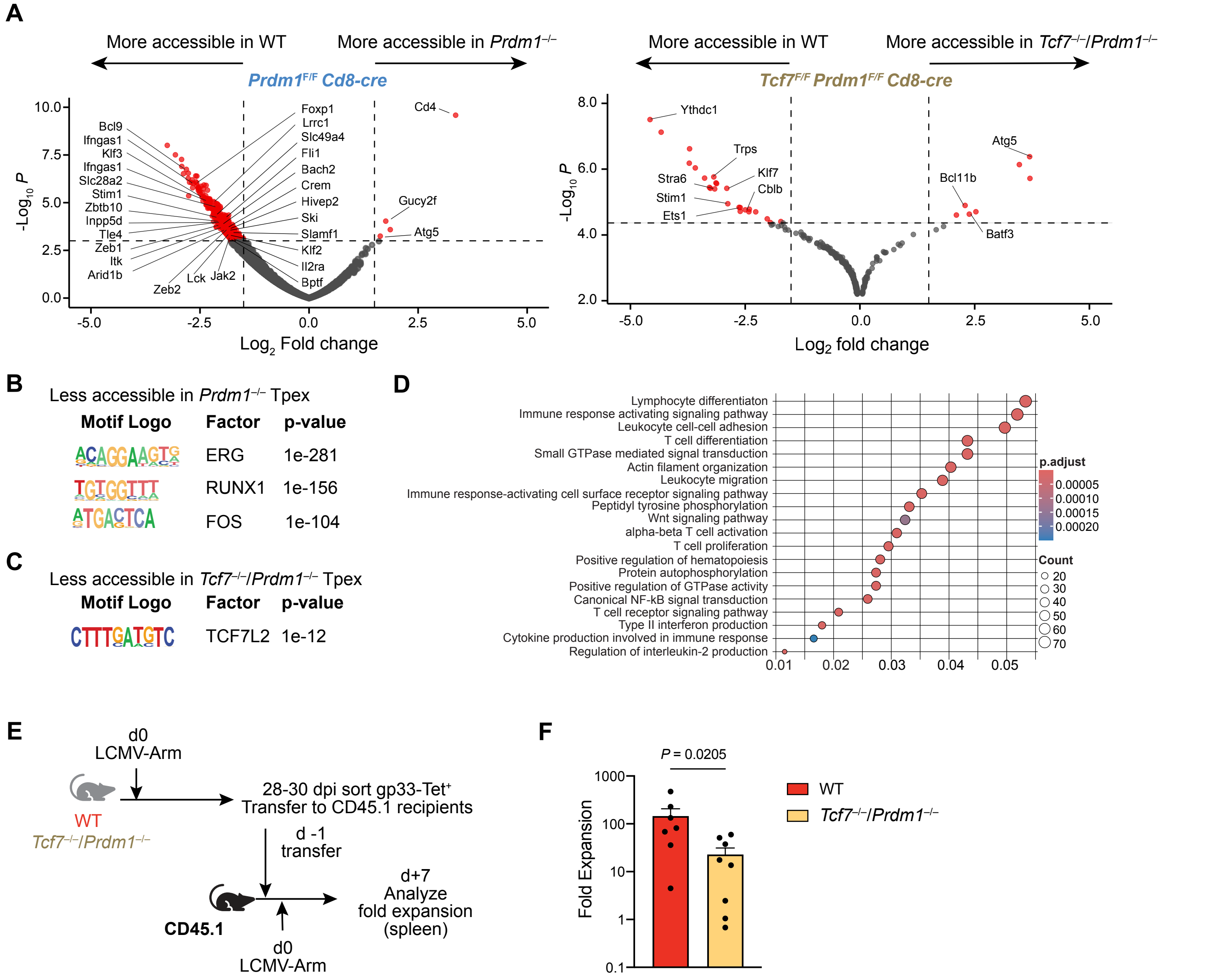

Figure S7. Epigenetic profiling of *Prdm1*$^{-/-}$ and *Tcf7*$^{-/-}$/*Prdm1*$^{-/-}$ Tpex and recall capacity of *Tcf7*$^{-/-}$/*Prdm1*$^{-/-}$ memory CD8$^+$ T cells, Related to Figure 7.

(A) Volcano plots of genes most proximal to differentially accessible chromatin regions in Tpex with indicated genotypes-compared to WT control. (B) *De novo* motif analysis by Homer for motifs enriched in DARs with reduced accessibility in *Prdm1*$^{-/-}$ than WT Tpex. (C) *De novo* motif analysis of DAR under-represented in *Tcf7*$^{-/-}$/*Prdm1*$^{-/-}$ than WT Tpex. (D) Gene ontology analysis of pathways differentially enriched in WT vs *Prdm1*$^{-/-}$ Tpex. (E) Experimental schematic corresponding to F. (F) Fold expansion of gp33-Tet$^+$ cells sorted from PBMC of WT and *Tcf7*$^{-/-}$/*Prdm1*$^{-/-}$ mice on 28-30 dpi with LCMV-Arm following transfer into naive recipients and rechallenge. Analyzed by Mann-Whitney test. Pooled from three independent experiments (n= 7 WT, 8 *Tcf7*$^{-/-}$/*Prdm1*$^{-/-}$).
